# Multiple, Distinct Intercontinental Lineages but Isolation of Australian Populations in a Cosmopolitan Lichen-Forming Fungal Taxon, *Psora decipiens* (Psoraceae, Ascomycota)

**DOI:** 10.3389/fmicb.2018.00283

**Published:** 2018-02-23

**Authors:** Steven D. Leavitt, Martin Westberg, Matthew P. Nelsen, John A. Elix, Einar Timdal, Mohammad Sohrabi, Larry L. St. Clair, Laura Williams, Mats Wedin, H. T. Lumbsch

**Affiliations:** ^1^Department of Biology and Monte L. Bean Life Science Museum, Brigham Young University, Provo, UT, United States; ^2^Museum of Evolution, Uppsala University, Uppsala, Sweden; ^3^Science and Education, The Field Museum, Chicago, IL, United States; ^4^Research School of Chemistry, Australian National University, Canberra, ACT, Australia; ^5^Natural History Museum, University of Oslo, Oslo, Norway; ^6^Department of Biotechnology, Iranian Research Organization for Science and Technology, Tehran, Iran; ^7^Plant Ecology and Systematics, Biology Institute, University of Kaiserslautern, Kaiserslautern, Germany; ^8^Department of Botany, Swedish Museum of Natural History, Stockholm, Sweden

**Keywords:** biogeography, biological soil crusts (BSC), cryptic species, disjunct populations, long-distance dispersal, *Psora*, semi-arid, South Africa

## Abstract

Multiple drivers shape the spatial distribution of species, including dispersal capacity, niche incumbency, climate variability, orographic barriers, and plate tectonics. However, biogeographic patterns of fungi commonly do not fit conventional expectations based on studies of animals and plants. Fungi, in general, are known to occur across exceedingly broad, intercontinental distributions, including some important components of biological soil crust communities (BSCs). However, molecular data often reveal unexpected biogeographic patterns in lichenized fungal species that are assumed to have cosmopolitan distributions. The lichen-forming fungal species *Psora decipiens* is found on all continents, except Antarctica and occurs in BSCs across diverse habitats, ranging from hot, arid deserts to alpine habitats. In order to better understand factors that shape population structure in cosmopolitan lichen-forming fungal species, we investigated biogeographic patterns in the cosmopolitan taxon *P. decipiens*, along with the closely related taxa *P. crenata* and *P. saviczii*. We generated a multi-locus sequence dataset based on a worldwide sampling of these taxa in order to reconstruct evolutionary relationships and explore phylogeographic patterns. Both *P. crenata* and *P. decipiens* were not recovered as monophyletic; and *P. saviczii* specimens were recovered as a monophyletic clade closely related to a number of lineages comprised of specimens representing *P. decipiens*. Striking phylogeographic patterns were observed for *P. crenata*, with populations from distinct geographic regions belonging to well-separated, monophyletic lineages. South African populations of *P. crenata* were further divided into well-supported sub-clades. While well-supported phylogenetic substructure was also observed for the nominal taxon *P. decipiens*, nearly all lineages were comprised of specimens collected from intercontinental populations. However, all Australian specimens representing *P. decipiens* were recovered within a single well-supported monophyletic clade consisting solely of Australian samples. Our study supports up to 10 candidate species-level lineages in *P. decipiens*, based on genealogical concordance and coalescent-based species delimitation analyses. Our results support the general pattern of the biogeographic isolation of lichen-forming fungal populations in Australia, even in cases where closely related congeners have documented intercontinental distributions. Our study has important implications for understanding factors influencing diversification and distributions of lichens associated with BSC.

## Introduction

Many symbiotic fungi have distinctive biogeographic patterns that do not conform with conventional expectations based on studies of animals and plants (MacArthur and Wilson, 1967; Galloway, 2008; Peay et al., 2010). Among symbiotic fungi, lichen-forming fungal lineages (Lücking et al., 2016b) are well known for unique and varied biogeographic patterns (Galloway, 2008; Werth, 2011). Species distributions of lichen-forming fungi range from truly widespread, intercontinental species with broad ecological amplitude (Wirtz et al., 2008; Fernández-Mendoza et al., 2011) to those with geographically and ecologically restricted distributions (Lücking et al., 2016a). While factors determining the establishment of populations of cosmopolitan species of lichen-forming fungi are not well understood, dynamic interactions among a variety of historical and ecological factors play important roles in determining species distributions (Leavitt and Lumbsch, 2016), e.g., reproductive strategies (Hilmo et al., 2012), availability of symbiotic partners (Fernández-Mendoza and Printzen, 2013; Werth and Sork, 2014), niche incumbency (Culberson and Culberson, 1967), and historical biogeography (Weber, 2003; Amo de Paz et al., 2012).

The origin of widely disjunct populations of lichen-forming fungi is not well understood for most lineages due to limited information on the temporal scale of divergences and population structure. Some species with disjunct populations may have had more contiguous distributions during the Tertiary period, with connections between North America and Asia, e.g., via Beringia or the North Pole by way of Greenland (Weber, 2003). Alternatively, intercontinental populations may be a result of long-distance dispersal events or migration into ecologically similar, disjunct habitats (Geml et al., 2012).

A number of cosmopolitan lichens occur in biological soil crust communities (BSCs) where they play important ecological roles (Büdel, 2003). The ecological importance of BSC communities is well-documented for arid and semiarid areas, the predominant environments for BSCs, which comprise approximately one third of the Earth’s total land area (Bowker et al., 2008). However, BSCs are also commonly found in temperate, subalpine, alpine, and nival zones (Hansen, 2003; Türk and Gärtner, 2003). In spite of the overall importance of lichens in BSCs and the well-documented degradation of these communities worldwide (Belnap et al., 2001), our understanding of the processes driving biogeographic and diversification patterns for soil crust lichens are poorly understood.

The lichen-forming fungal species *Psora decipiens* (Hedwig) Hoffm. is a common component of BSCs worldwide and occurs in habitats ranging from hot, arid deserts to alpine tundra/steppes (Timdal, 1984). The ability of *P. decipiens* to form symbiotic relationships with a broad range of photosynthetic algal partners, including multiple lineages within *Asterochloris* and *Trebouxia*, has been proposed as an important factor contributing to the successful establishment of this lichen across diverse habitats (Ruprecht et al., 2014). However, conflicting results suggested that the range of suitable photosynthetic partners for *P. decipiens* may actually be restricted to only two distinct clades in the green algal genus *Myrmecia* (Geitler, 1963; Williams et al., 2017). European populations of *P. decipiens* are comprised of distinct genotypes apparently adapted to specific climactic conditions, which are likely unable to acclimate to the different environmental conditions found within the species range or switch photobiont partners across short time scales (Williams et al., 2017). However, genetic diversity in populations outside of Europe have not been well characterized.

While the occurrence of *P. decipiens* as disjunct populations in Asia, Europe, and North America may be an artifact of much broader distribution predating the Quaternary (Weber, 2003), the origin of populations in Australia are particularly enigmatic. Australia includes one of the largest arid biomes in the world, supporting a unique, diverse and relatively well-studied biota (Byrne et al., 2008), although the impact of the isolation and aridification of Australia on symbiotic fungal radiations remains largely unexplored. From a biogeographic perspective, lichen-forming fungi in Australia are, in general, well-separated from their closest related congeners (Argüello et al., 2007; Elix et al., 2009; Amo de Paz et al., 2012). The physical origin of Australia’s desert biome is generally well characterized and includes three major events: (i) the rifting of Australia from Antarctica (accelerated ca. 32 Ma); (ii) the advent of the Antarctic Circumpolar Current; and (iii) the onset of severe aridity during the Pliocene (Crisp et al., 2004). The tectonic isolation of Australia led to a transition from an aseasonal-wet biome to the unique Australian sclerophyll biomes dominated by eucalypts, acacias, and casuarinas, accompanied by another major shift in the Australian biota during the late Oligocene and Miocene (Crisp et al., 2004). Dispersal of *P. decipiens* to Australia likely occurred via long distance dispersal by spores, and BSC-type habitats suitable for *P. decipiens* may not have been present in Australia before the Pliocene.

*Psora decipiens* appears to belong to a group of closely related soil dwelling lichens, including *P. crenata* (Taylor) Reinke and *P. saviczii* (Tomin) Follmann & A. Crespo. Generally, *P. crenata* is separated from *P. decipiens* by the presence of larger squamules with a prominent central depression and the presence of the extrolite norstictic acid. However, morphological and chemical variation in *P. decipiens* is well known (Timdal, 1984), including populations of *P. decipiens* with norstictic acid in Australia, the Mediterranean, and some Arctic localities. *P. saviczii* is morphologically similar to *P. decipiens* but differs largely based on its occurrence on gypsum-based soils in central Spain, where it has been considered a threatened lichen as defined by IUCN categories (Atienza and Segarra, 2000). Given the absence of fixed diagnostic characters separating these taxa and overlapping geographic and ecological distributions, an alternative view is that *P. crenata, P. decipiens*, and *P. saviczii* should be considered a single variable species.

In order to better understand the biogeographic and diversification patterns of cosmopolitan BSC lichens, we sampled *Psora* specimens (*P. crenata, P. decipiens*, and *P. saviczii*) from worldwide populations in Australia, Central Asia, Europe, the Middle East, North America, and South Africa. From this sampling, we generated a multi-locus sequence dataset which we used to reconstruct evolutionary relationships for these three taxa. Our specific research questions were: (i) What are the phylogenetic relationships among *P. crenata, P. decipiens*, and *P. saviczii*? (ii) Are *P. decipiens* populations structured phylogeographically? (iii) How isolated are Australian populations from other *P. decipiens* populations? and (iv) Can we place the diversification history of this group within a temporal context?

## Materials and Methods

### Taxon Sampling

**FIGURE 1:**
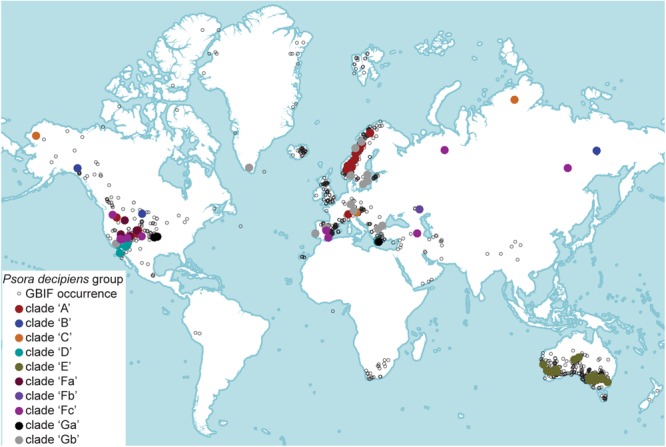
The colored, filled circles represent the candidate species. Geographic distribution of *Psora decipiens* s. lat. Empty, black circles represent records from Global Biodiversity Information Facility (GBIF; https://www.gbif.org). Colored circles represent specimens included in the present study, and different colors correspond to the distinct candidate species circumscribed using molecular sequence data.

Our sampling focused on the cosmopolitan lichen-forming taxon *P. decipiens*, and closely related species *P. crenata* and *P. saviczii*; and a total of 159 specimens were included (**Supplementary Table S1**). *Psora crenata* and *P. decipiens* are broadly distributed, and specimens were opportunistically sampled with the intent to represented a wide range of populations dispersed across broad geographic and ecological distributions. Our sampling of *P. crenata* included 25 specimens: three from western North America, one from the Middle East (Yemen), and 21 from South Africa. *Psora decipiens* was represented by 129 specimens, including 16 from Asia [Iran (11); Russia (5)], 35 from Australia, 57 from Europe, and 25 from North America (**Figure 1**). Specimens representing *P. saviczii* were sampled from Spain (3) and Russia (1). Latitude and longitude were used to extract bioclimatic variables at a spatial resolution of 2.5 min, using the R (R Core Team, 2015) package raster (Hijmans et al., 2015). All specimens were examined using an Olympus SZH stereomicroscope, and, for selected specimens, thin-layer chromatography (TLC) was performed following the methods of Culberson C.F. (1972), as modified by Menlove (1974) and Culberson and Johnson (1982). A single specimen representing *P. tuckermanii* R. Anderson ex Timdal was selected as the outgroup based on Ekman and Blaalid (2011) and exploratory analyses.

#### DNA Extraction, Amplification, and Sequencing

Total genomic DNA was extracted from specimens using the USB PrepEase Genomic DNA Kit (Affymetrix, Santa Clara, CA, United States – product discontinued), DNeasy Plant MiniKit (Qiagen), or the ZR fungal/bacterial DNA miniprep kit (Zymo Research). We generated molecular sequence data for a total of five markers: two nuclear ribosomal loci were sampled, including the large-subunit (nuLSU) and the internal transcribed spacer region (ITS – including ITS1, 5.8S, and ITS2); a fragment of the mitochondrial small subunit (mtSSU); and fragments from two protein-coding loci, the mini-chromosome maintenance complex component 7 (*MCM7*), and RNA polymerase II largest subunit (*RPB1*). Sequences were generated using Sanger sequencing, and primers and conditions for polymerase chain reaction (PCR) amplifications for all loci follow previous studies (Blanco et al., 2004; Schmitt et al., 2009; Leavitt et al., 2013b). PCR amplifications were performed using Ready-To-Go PCR Beads (GE Healthcare, Pittsburgh, PA, United States), with cycling parameters following a 55–50°C touchdown reaction (Lindblom and Ekman, 2006). PCR products were visualized on 1% agarose gel and cleaned using ExoSAP-IT (USB, Cleveland, OH, United States). Complementary strands were sequenced using the same primers used for amplifications, and sequencing reactions were performed using BigDye 3.1 (Applied Biosystems, Foster City, CA, United States). Products were run on an automated sequencer (ABI Prism 377) located in the Molecular Systematic Laboratory at the Swedish Museum of Natural History in Stockholm or an ABI 3730 automated sequencer (Applied Biosystems) at the Pritzker Laboratory for Molecular Systematics and Evolution at the Field Museum, Chicago, IL, United States and the DNA Sequencing Center at Brigham Young University, Provo, UT, United States.

### Sequences Assembly, Multiple Sequence Alignments and Estimating Substitution Models

Bi-directional Sanger reads were assembled and edited using the program Sequencher 4.10 (Gene Codes Corporation, Ann Arbor, MI, United States), and sequences were aligned using the program MAFFT 7 (Katoh et al., 2005; Katoh and Toh, 2008). We specified the G-INS-i alignment algorithm and ‘1PAM/K = 2’ scoring matrix, with an offset value of 0.9. The remaining parameters were set to default values for the protein-coding (*MCM7* and *RPB2*) and nuLSU markers. For the mtSSU, we used the same parameters, with the exception of an offset value set to 0.1 rather than 0.9 and ‘unalignlevel’ set to 0.6. Exploratory multiple sequence alignments of the ITS resulted in a substantial proportion of ambiguously aligned regions across the *P. crenata, P. decipiens*, and *P. saviczii* groups. Therefore, the ITS data was only aligned for the *P. decipiens* group, including *P. saviczii*. Each of subclade within the *P. decipiens* group (see Results) was aligned individually using the G-INS-I and ‘1PAM/K = 2’ scoring matrix, with an offset value of 0.1, and the remaining parameters were set to default values for the protein-coding. Individual sub-alignments were merged using MAFFT using the same parameters as described above for each individual sub-alignment. For the Bayesian analysis (see below), substitution models for each locus were estimated using jModelTest v.2.1.10 (Darriba et al., 2012).

### Phylogenetic Analyses and Divergence Time Estimates

Phylogenetic relationships among the sampled fungal lineages were inferred using both maximum likelihood (ML) and Bayesian inference (BI) methods. Due to ambiguities in an exploratory ITS alignment across all samples, ML topologies were inferred from two concatenated multi-locus data matrices: (1) a four-locus matrix comprised of the nuLSU, *MCM7, RPB1*, and mtSSU sequences representing the complete taxon sampling – *P. tuckermanii* (outgroup), *P. crenata, P. decipiens*, and *P. saviczii*; and (2) a five-locus matrix comprised of the ITS, nuLSU, *MCM7, RPB1*, and mtSSU alignments for the *P. decipiens* and *P. saviczii* samples only (outgroup and *P. crenata* excluded). ML phylogenies were inferred using the program RAxML v8.2.1 (Stamatakis, 2006; Stamatakis et al., 2008) in the CIPRES Science Gateway server^1^, the data matrices were partitioned by locus, and applying the GTRGAMMA’ model. Nodal support was evaluated using 1000 bootstrap pseudo-replicates.

We used the program BEAST v1.8.3 (Drummond and Rambaut, 2007; Heled and Drummond, 2010) to reconstruction relationships within a Bayesian framework. Relevant fossil and rate calibrations are not available for Psoraceae. However, as a hypothesis-generating approach, we used a substitution rate for the *RPB1* estimated for the entire Lecanorales, 1.65 substitutions/site/year X^-9^ (Amo de Paz et al., 2011), to place the diversification of the *P. decipiens* group within a temporal context. Bayesian topologies were inferred from the two concatenated multi-locus data matrices defined above – the four marker dataset representing the complete taxon sampling and the five-locus dataset representing the focal group, *P. decipiens* and *P. saviczii*. Substitution rates were co-estimated for each locus under a uniform prior and relative to the fixed 1.65 substitutions/site/year X^-9^ rate for the *RPB1*. The BI analyses were performed using a Yule speciation process prior and with the data matrix partitioned by individual gene regions, and applying the locus-specific models inferred using jModelTest (see section “Results”). Divergence times were estimated under a strict molecular clock (Drummond et al., 2006), and two independent MCMC runs of 25 million generations were performed, sampling every 1000 steps. Chain mixing and convergence were evaluated in Tracer v1.6 (Rambaut and Drummond, 2003), considering ESS values > 200 as good indicators. After excluding the first 25% of sampled trees as burn-in, trees from the two independent runs were combined using the program LogCombiner v1.8.3 (Rambaut and Drummond, 2013), and the final MCC tree was estimated from the combined posterior distribution of trees using TreeAnnotator v1.8.3 (Rambaut and Drummond, 2009).

### Delimiting Candidate Species

Well-supported phylogenetic substructure was inferred from the multi-locus data matrices, and we treated distinct, well-supported clades within the nominal species *P. crenata* and *P. decipiens* as candidate species-level lineages (see section “Results”). For the *P. decipiens* group, which included *P. saviczii*, relationships of candidate species were evaluated among individual ITS, *MCM7*, and *RPB1* topologies to identify lineages that exhibited genealogical exclusivity across multiple loci (Avise and Ball, 1990; Hudson and Coyne, 2002). The presence of the same clades in the majority of single-locus phylogenies is taken as evidence that the clades represent reproductively isolated lineages (Dettman et al., 2003; Pringle et al., 2005). Genealogical concordance provides a conservative approach for robustly delimiting species boundaries among putative lineages, although it may fail to delimit species with more recent diversification histories due to incomplete lineage sorting (Leavitt et al., 2016a). Individual locus topologies were inferred using the program RAxML v8.2.1 in the CIPRES Science Gateway server^2^. Nodal support was evaluated using 1000 bootstrap pseudo-replicates.

The evolutionary independence of the 10 candidate species representing *P. decipiens* was validated using program BPP v3.2 (Yang and Rannala, 2010, 2014; Rannala and Yang, 2013). We used the unguided species delimitation analysis ‘A11’ (Yang, 2015), which explores different species delimitation models and different species phylogenies, with fixed specimen assignments to populations. Analysis ‘A00’ (Yang, 2015), a within-model inference, was used to generate the posterior distribution of the parameters theta (𝜃s) and tau (τs) under the multispecies coalescent model (MSC) model to infer a reasonable combination of priors given the data (Rannala, 2015). Based on the results from the ‘A00’ analyses, the gamma prior G for 𝜃 was set to ∼G(1,125), and the gamma prior G for tau (τ) was set to ∼G(1,300). Under the unguided species delimitation model, ‘A11,’ we used two different search algorithms (algorithm 0 or 1), with equal probabilities for the labeled histories, to assign probabilities to the models, rates were allowed to vary among loci (locus rate = 1), and the analyses were set for automatic fine-tune adjustments. The rjMCMC analysis was run for 100,000 generations, sampling every two generations and discarding the first 10% as burn-in. The analysis was run twice to confirm consistency between runs.

### Niche Data Comparisons among Candidate Species

Individual bioclimatic variables were compared among candidate species using boxplots. Data are provided in SI. Additionally, a phylogenetic PCA was performed to help visualize each candidate species distribution in multidimensional niche space. The four-gene phylogeny was reduced to include a single representative of each candidate species, and the mean value for each bioclimatic variable was obtained for each candidate species. A phylogenetic PCA using the correlation matrix was then run in the R package phytools (Revell, 2012).

## Results

### Molecular Sequence Data

New sequences generated in association with this study have been deposited in GenBank under accession numbers MG677156–MG677545; MG783593–MG783845. Both data matrices – the complete taxon sampling (nuLSU, *MCM7, RPB1*, and mtSSU) and the *P. decipiens* group (ITS, nuLSU, *MCM7, RPB1*, and mtSSU) – and associated tree files were submitted to TreeBase (submission ID: 20946). Locus-specific substitution models inferred using jModelTest were: nuLSU, GTR+I+G; ITS, SYM+I+G; *MCM7*, TrNef+I; *RPB1*, SYM+G; and mtSSU, TPM1uf.

### Phylogenetic Relationships and Estimated Divergence Times

**FIGURE 2:**
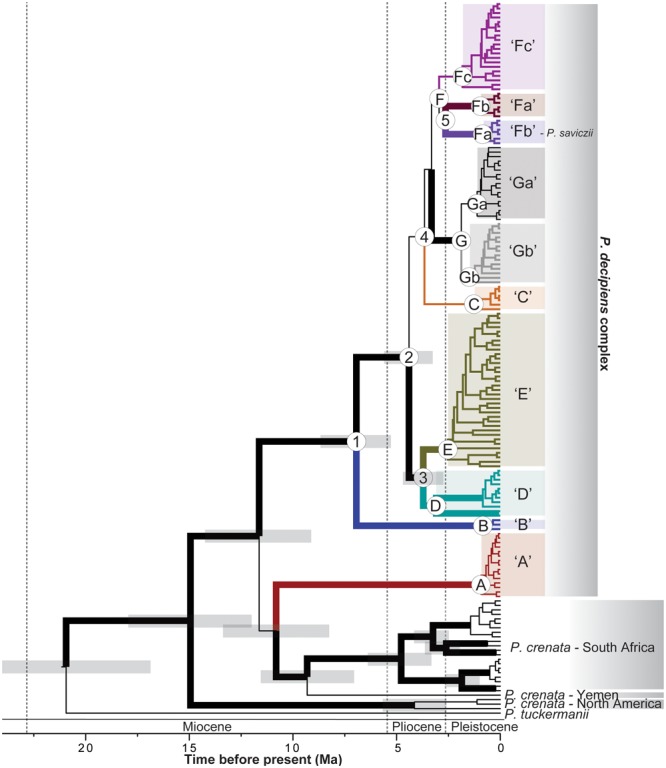
A rate-calibrated chronogram for the *P. crenata, P. decipiens*, and *P. saviczii* clade. The topology was inferred from a four-marker (nuLSU, *MCM7, RPB1*, and mtSSU) data matrix using the program BEAST; and divergence times were estimated using a fixed rate for the *RPB1* locus (1.65 substitutions/site/year X^-9^) previously estimated for the lichen-forming fungal order Lecanorales. The Oligocene-Miocene (O/M), Miocene-Pliocene (M/P), and Pliocene-Pleistocene (P/P) boundaries are indicated by dashed vertical lines. Posterior probabilities ≥ 0.95 are indicated with thickened branches. Candidate species from the *P. decipiens* complex are indicated by distinct colors and codes (‘A’–‘Gb’) and correspond to identical colors in all other figures. Number nodes are referred to in the text.

**FIGURE 3:**
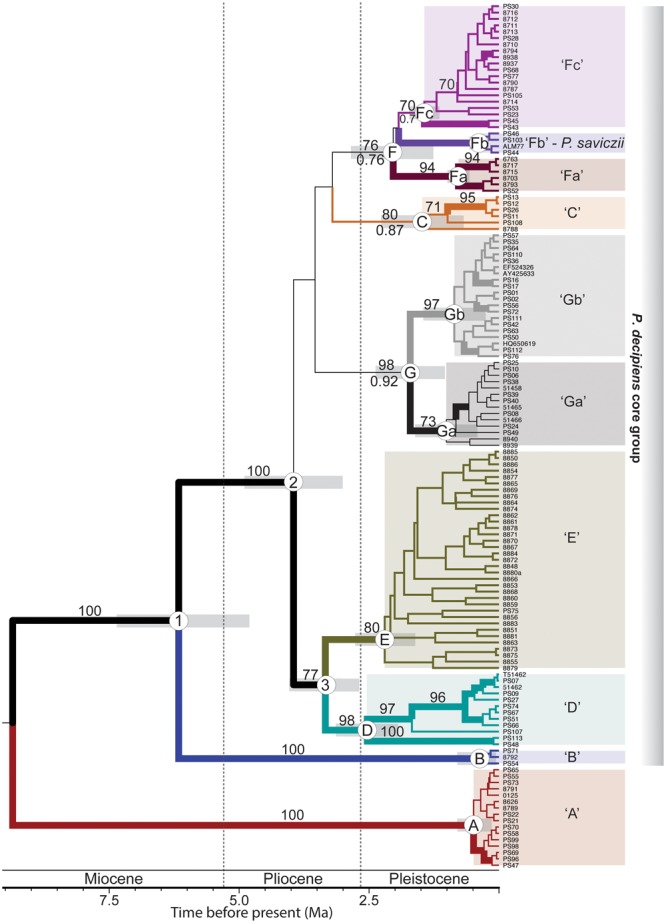
A rate-calibrated chronogram for the *P. decipiens* complex. The topology was inferred from a five-marker (ITS, nuLSU, *MCM7, RPB1*, and mtSSU) data matrix using the program BEAST; and divergence times were estimated using a fixed rate for the *RPB1* locus (1.65 substitutions/site/year X^-9^) previously estimated for the lichen-forming fungal order Lecanorales. The Miocene-Pliocene and Pliocene-Pleistocene boundaries are indicated by dashed vertical lines. Posterior probabilities ≥ 0.95 are indicated with thickened branches; and maximum likelihood bootstrap is indicated above the branches. Candidate species from the *P. decipiens* complex are indicated by distinct colors and codes (‘A’–‘Gb’) and correspond to identical colors in all other figures. Number nodes are referred to in the text.

Both the ML and BI phylogenetic reconstructions resulted in similar topologies, providing a generally well-resolved backbone for the *P. crenata*/*P. decipiens* clade (**Figure 2**). Neither *P. crenata* nor *P. decipiens* were recovered as monophyletic in analyses of the complete data matrix (**Figure 2**); and *P. saviczii* (clade ‘Fb’) was recovered as a monophyletic clade nested among a number of clades comprised of *P. decipiens* specimens (**Figure 2**). *P. crenata* specimens were recovered in three, deeply diverging clades, each corresponding to the three distinct geographic regions sampled for this study: The Middle East (Yemen; one sample), South Africa, and western North America (**Figure 2**). Furthermore, well-supported phylogenetic substructure was reconstructed for *P. crenata* specimens collected from South Africa, but with no clear regional biogeographic structuring. Phylogenetic reconstructions of the complete taxon sampling revealed distinct lineages comprised of *P. decipiens* samples (**Figure 2**), and seven well-supported clades – ‘A’ through ‘G’– were recovered in analyses of the five-locus dataset representing the *P. decipiens* core group (**Figure 3**). Clade ‘F’ was further divided into three subclades, ‘Fa,’ ‘Fb,’ and ‘Fc,’ with clade ‘Fb’ comprised exclusively of all *P. saviczii* specimens. Two subclades were also recognized in clade ‘G,’ ‘Ga,’ and ‘Gb’ (**Figure 3**).

Each clade representing the *P. decipiens* core group included specimens collected from intercontinental distributions, with the exception of clade ‘E,’ which was comprised exclusively of Australian specimens (**Figure 1** and **Table 1**). Confirmed geographic distributions and habitat preferences for each clade are summarized in **Table 1**. Specimens recovered in clades ‘A’ and ‘C’ were collected from high altitude/latitude populations in Europe, North America, and Russia, occurring in regions with generally lower temperatures and higher precipitation than those from the other clades (**Supplementary Figure S1**). However, no bioclimatic variable clearly demarked the ecological preferences of the other clades, although some differences are identifiable at regional scales. For example, the Scandinavian *P. decipiens* specimens were recovered in two distinct clades: one found in inland populations (clade ‘A’) and the other occurring in more coastal soil crust communities (clade ‘Gb’). Other intercontinental populations of *P. decipiens* were also recovered in both clades ‘A’ and ‘Gb,’ and not all specimens recovered in clade ‘Gb’ were collected from soil crust communities with a maritime influence, e.g., populations in Germany and Southern California, United States (**Figure 1** and **Supplementary Table S1**). TLC results revealed the presence of multiple chemotypes in a number of *P. decipiens* lineages (clades ‘D,’ ‘E,’ ‘Fc,’ ‘Ga,’ and ‘Gb’), with Australian specimens, clade ‘E,’ harboring the highest extrolite diversity. No compounds were detected in the sampled specimens recovered in clades ‘A,’ ‘B,’ ‘C,’ ‘Fa,’ and ‘Fb’ (*P. saviczii*) (**Table 1** and **Supplementary Table S1**).

**Table 1:** Generalized information summarized for candidate species circumscribed in this study, including geographic distributions, habitat preferences, and secondary metabolites.

Candidate species	Geography distribution	Habitat	Secondary metabolites
‘A’ (*n* = 16)	Italy, Norway, Sweden, and United States (CO, OR, UT)	Alpine/subalpine habitats with relatively low temperatures and high precipitation in western United States and the Italian Alps; inland habitats in Scandinavia	None detected
‘B’ (*n* = 3)	Canada (YT), Russia (Yakutia), and United States (ND)	Cold, dry high latitude habitats in Asia and North America.	None detected
‘C’ (*n* = 6)	Austria (Austrian Alps), Russia (Taymyr Peninsula), and United States (Alaskan North Slope)	High latitude sites with relatively low temperatures and high precipitation in Asia and North America; alpine habitat in the Austrian Alps.	None detected
‘D’ (*n* = 11)	Greece, Mexico (Baja California), Portugal, Russia (Sahka), Spain, and United States (AZ, CA)	Hot, dry continental habitats in western North America, Europe, and Asia.	Not detected; norstictic acid (major)
‘E’ (*n* = 35)	Australia	Arid Australia drylands	Not detected; norstictic acid (major); norstictic acid (major) and conorstictic acid (trace); 3α-hydroxy-4-*O-*demethylbarbatic (major) and 3α-hydroxynorobtusatic (minor); hypoprotocetraric (major) and 4-O-demethylnotatic (minor); and hypoprotocetraric (major);
‘Fa’ (*n* = 6)	Iran and United States (CO, ID, NV, UT)	Northern Iran and sage-steppe communities in western North America; on calciferous to gypsiferous soils	None detected
‘Fb’ - *P. saviczii* (*n* = 4)	Spain and Russia (Astrakhan Oblast)	Hot, dry, gypsiferous habitats in Spain and Russia	None detected
‘Fc’ (*n* = 20)	Iran, Russia (Komi), Spain, and United States (AZ, CO, KS, NV, UT, WA)	on calciferous to gypsiferous soils in Central Asia, Europe, and North America	Not detected; hyposalazinic and hypostictic
‘Ga’	Greece, Spain, and United States (KS)	Calciferous to gypsiferous soils (Spanish specimens) in Europe and Midwestern United States	Not detected; norstictic
‘Gb’	Bulgaria, Germany, Greece, Greenland, Norway, Portugal, Sweden, and United States (CA)	Generally, from soil crust communities with a maritime influence, with the exception of samples from Germany (specimens ‘PS01’ and ‘PS02’) and southern California, United States (specimen ‘P76’).	Not detected; norstictic

Our estimates suggest a most recent common ancestor (MRCA) for the *P. crenata*/*P. decipiens* clade at ca. 15 Ma [95% Highest Posterior Density (HPD) = 12.1–18.1 Ma] and a MRCA for the core *P. decipiens* group at ca. 7 Ma (95% HPD = 5.4–8.7 Ma) (node ‘1,’ **Figure 2**). Our estimates of divergence times for the *P. crenata*/*P. decipiens* clade support a Miocene-dominated diversification history for the major lineages within this group and a Pliocene-dominated history for the core group of *P. decipiens* lineages (clades ‘B’ – ‘G’) (**Figure 2**). Similar divergent times were inferred from the 5-locus matrix restricted to the *P. decipiens* core group, which included the ITS, nuLSU, *MCM7, RPB1*, and mtSSU alignments (**Figure 3**). The MRCA for each candidate species-level lineage circumscribed here most likely occurred in the Quaternary (**Figure 3**). For the analysis of the *P. decipiens* core group, substitution rates co-estimated relative to the 1.65 substitutions/site/year X^-9^ for the *RPB1* locus were: nuLSU = 1.22 X^-9^ s/s/y (95% HPD interval = 0.83–1.65 X^-9^ s/s/y); ITS = 5.38 X^-9^ s/s/y (95% HPD interval = 4.11–6.82 X^-9^ s/s/y); *MCM7* = 2.27 X^-9^ s/s/y (95% HPD interval = 1.63–2.97 X^-9^ s/s/y); and mtSSU = 0.31 X^-9^ s/s/y (95% HPD interval = 0.16–0.48 X^-9-9^ s/s/y).

### Candidate Species

**FIGURE 4:**
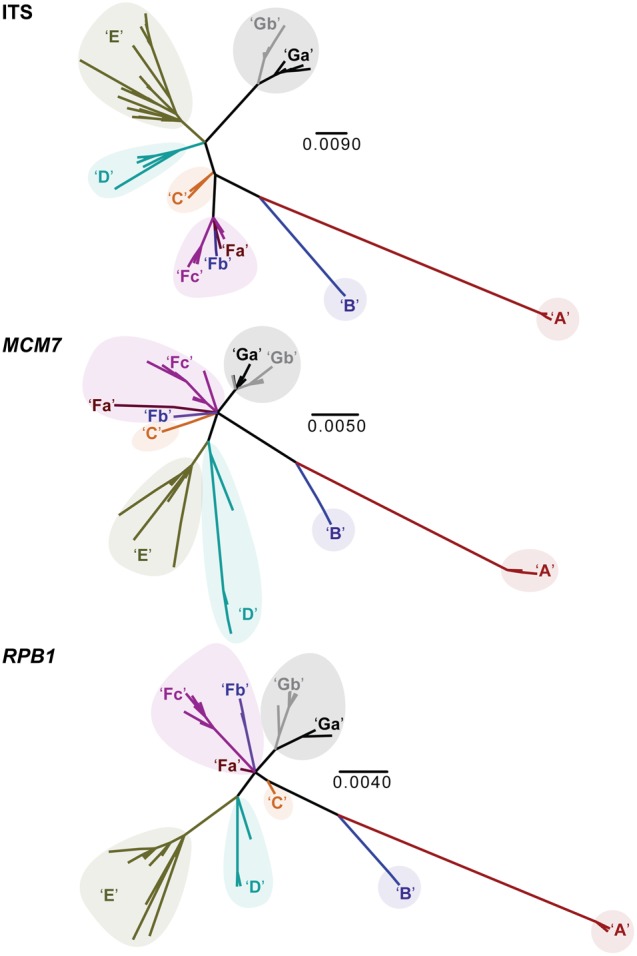
Unrooted individual locus topologies for the ITS, *MCM7*, and *RPB1* markers. Candidate species are indicated by color and code (‘A’–‘Gb’), corresponding to identical colors in all other figures. Scale bar in each panel represents substitutions per site.

Individual locus topologies showed a general pattern of genealogical concordance for the 10-candidate species in the *P. decipiens* core group that were initially delimited based on well-supported phylogenetic structure (**Figure 4**). These species-level lineages were further supported by high speciation probabilities (>0.99) in the coalescent-based validation method BPP. All candidate species delimited in the *P. decipiens* core group reflected high genetic variability, with the exception of clade ‘A’ (**Table 2**).

**Table 2:** Polymorphism statistics for the internal transcribed spacer region (ITS) in candidate species within the *P. decipiens* complex.

Clade	*N*/*S*/*Hd*	π
‘A’ (522)	16/4/0.70	0.00153
‘B’ (583)	3/2/0.67	0.00229
‘C’	5/8/0.70	0.00561
‘D’	11/23/0.93	0.01486
‘E’	29/53/0.97	0.01922
‘Fa’	5/3/0.60	0.00499
‘Fb’ – *P. saviczii*	4/1/0.50	0.00087
‘Fc’	18/15/0.882	0.00882
‘Ga’	14/14/0.835	0.00423
‘Gb’	20/13/0.826	0.00676

*N, number of individuals sampled; S, number of polymorphic sites; Hd, haplotype diversity; π, estimate of 4 Nl per base pair using the average pairwise differences*.

## Discussion

Characterizing evolutionary processes and biogeographic patterns in widely distributed components of BSCs has important implications, ranging from management and conservation to biodiversity inventories to providing an improved framework for ecological and evolutionary research. Our study of the cosmopolitan lichen-forming taxon *P. decipiens*, and closely related species *P. crenata* and *P. saviczii*, reveals complex biogeographic patterns, previously unrecognized species-level diversity, and a diversification history spanning millions of years for this important clade of biological soil crust lichens. Phylogenies inferred from multi-locus sequence data provide a generally well-supported hypothesis of relationships and novel insight into diversity and distributions of the sampled taxa and worldwide populations (**Figure 2**). Neither *P. crenata* nor *P. decipiens* were recovered as monophyletic and multiple species-level lineages were circumscribed in both nominal taxa based on molecular data. Strikingly, our results revealed multiple widespread, intercontinental candidate species comprised of *P. decipiens* specimens, in contrast to geographically restricted species-level lineages representing *P. crenata* populations. However, all Australian populations representing the nominal taxon *P. decipiens* belonged to a singled species-level clade that was not sampled outside of Australia. Below we discuss the implications of our study for better understanding the diversification of lichen-forming fungi in BSCs commonly associated with and arid and semi-arid habitats.

### Contrasting Patterns of Geographically Restricted and Intercontinental Species-Level Lineages

Floristic similarities in widely disjunct geographic regions have long fascinated biologists and are well-known in lichen-forming fungi (Culberson W.L., 1972; Galloway, 2008). However, due to limited information on genetic population structure and the temporal scale of divergences, the origin of widely disjunct populations remains unclear for most cosmopolitan species. Two general explanations of disjunct lichen distributions prevail: (1) species with disjunct populations had more contiguous distributions during the Tertiary period; or the alternative explanation that (2) disjunct populations are a result of long-distance dispersal events or migration into ecologically similar, disjunct regions (Weber, 2003). For example, in the case of the former, Weber (2003) characterized the Rocky Mountain flora as “a microcosm of its rich Middle Asiatic counterpart,” with the present disjunctions representing relict populations of a once widely distributed Oroboreal flora. However, more recent studies suggest a more nuanced perspective into the temporal and spatial components of diversification in cosmopolitan lichen-forming fungi species, highlighting the role of long-distance dispersal during the Quaternary for many species (Buschbom, 2007; Geml et al., 2010; Otálora et al., 2010; Fernández-Mendoza and Printzen, 2013; Leavitt et al., 2013a).

Our study of *P. decipiens*, based on data from worldwide populations, also supports the idea of effective long-distance dispersal by soil crust lichens. In fact, nine of the 10 candidate species circumscribed from our cosmopolitan sampling of *P. decipiens* s. lat. occurred in disjunct, intercontinental populations (**Table 1**). While the MRCA for the core *P. decipiens* group was estimated to be during the late Miocene (Node ‘1,’ **Figure 3**), the MRCA for specimens comprising each putative species within this group was inferred to have occurred during the Pleistocene. If disjunct *P. decipiens* populations were relicts of past continuous distributions predating the Pleistocene we would expect to observe biogeographically structured clades with deeper divergence times, rather than intercontinental lineages with similar haplotypes shared across disjunct populations. In spite of the capacity of *P. decipiens* s. lat. for effective long-distance dispersal across Asia, Europe and North America, our results suggest all extant populations in Australia resulted from only a single dispersal event. The Australian clade, clade ‘E,’ shares a MRCA with its sister clade, clade ‘D’ during the mid-Pliocene (node ‘3,’ **Figure 3**), a time of increasing aridity worldwide (Crisp et al., 2004). In southwestern Australia, the prevailing mild wet climate that prevailed into the Pliocene was replaced by the extreme wet–dry glacial cycles typical of the present climatic system that began approximately 2.9 Ma (Dodson and Macphail, 2004); likely resulting in suitable habitat and environmental conditions for successful establishment of BSC communities.

In contrast to the core *P. decipiens* group, *P. crenata* s. lat. populations showed strong phylogeographic patterns, with populations in North American, South Africa, and The Middle East forming three distinct lineages, with deeper divergence times than the core *P. decipiens* group (**Figure 2**). In spite of the widespread, intercontinental distributions of many lichens species, South African and Australian populations of nominal taxa with intercontinental distributions are commonly found to belong to distinct evolutionary lineages (Argüello et al., 2007; Hodkinson and Lendemer, 2011). While we were unable to obtain fresh specimens representing *P. crenata* from Australia, we would predict that these specimens would likely be recovered as a fourth major lineage representing this nominal taxon.

Both global cooling and mountain uplift have been proposed as playing important roles in the long-term aridification during the Mid–Late Miocene (Miao et al., 2012), and our divergence estimates for the *P. crenata*/*P. decipiens* clade generally coincide with this pattern of aridification (**Figure 2**). The Miocene also appears to be a major epoch of diversifications for other lineages of lichen-forming fungi, including many lineages in the hyper-diverse lichen-forming fungal family Parmeliaceae (Amo de Paz et al., 2011; Kraichak et al., 2015), as well as others in Lecanoraceae (Leavitt et al., 2016b). Our results suggest that the expansion of open habitats during the last 5 million years (Cerling et al., 1997; Fortelius et al., 2006) played a major role in diversification in the *P. decipiens* core group (**Figure 3**). Similar patterns have been observed for other organisms adapted to open habitats, including falcons (Fuchs et al., 2015), C_4_ grasslands (Edwards et al., 2010), other lichen-forming fungi (Amo de Paz et al., 2012; Leavitt et al., 2013b), and humans (Bobe and Behrensmeyer, 2004).

In spite of the interesting correlations between major global climatic shifts and divergence times estimated for the *P. crenata*/*P. decipiens* clades, we emphasize that our divergence estimates must be interpreted with caution. There are significant limitations to the rate-calibrated approach implemented here and based on an order-wide estimate for a single locus (Amo de Paz et al., 2011). While recent studies highlight the promise of incorporating fossil evidence into divergence dating of lichen-forming fungi (Divakar et al., 2015; Kaasalainen et al., 2015), to our knowledge, no relevant fossil evidence is available for the placing the diversification of the genus *Psora* into a temporal framework. Similarly, more accurate estimates of substitution rates are not available for Psoraceae. Therefore, we strongly caution against over-interpreting our divergence estimates and only include them as a hypothesis-generating approach for considering the temporal context of diversification in this group.

### Potential for Ecological Specialization in the *Psora decipiens* Core Group

The results of this study indicate that *P. decipiens* specimens occurring across diverse ecological habitats are commonly found in distinct, species-level clades (**Table 1**). In some cases, ecological differences, rather than geographic proximity may be a better predictor of clade membership. Regions with ecologically diverse habitats, such as the southwestern portion of the United States, harbor a wide range candidate species, while populations that are isolated across thousands of kilometers may belong to a single species-level lineage, e.g., geographically isolated populations in Russia belong to the same species-level lineage ‘Fc’ (**Figure 1**). However, based on limitations of our current sampling (small sample sizes for a number of lineages, limited environmental data at collecting sites, etc.), robustly characterizing ecological differences among the candidate species is beyond the scope of this study. Results from previously published studies and the evidence presented here highlight the importance of additional research into ecological specialization in the *P. decipiens* group.

Williams et al. (2017) identified four distinct clades representing European populations of *P. decipiens* and demonstrated that different genotypes were unable to acclimate to different environmental conditions within the species’ range. Additional evidence suggests that differences among these clades may be related to the regulation of thallus water content, with some clades specifically adapted to conditions found in semi-arid habitats, while others are better adapted to seasonally wet, alpine habitats (Colesie et al., 2017). Our additional sampling supports this distinction. Specifically, clade ‘C’ (this study) corresponds to the ‘Austria’ clade [Figure 5 in Williams et al. (2017)]; and all specimens comprising this clade were collected for alpine sites characterized by relatively lower temperatures and higher precipitation (**Supplementary Figure S1**). However, our sampling also revealed another alpine-adapted lineage of *P. decipiens* s. lat, clade ‘A’ (**Figure 3** and **Supplementary Figure S1**), with a somewhat different geographic distribution relative to clade ‘C’ (**Figure 1** and **Table 1**). A third clade appears to be restricted to relatively cold habitats in North America, clade ‘B,’ but differs from clades ‘A’ and ‘C’ by its occurrence in habitats with much lower precipitation (**Supplementary Figure S1**). Additional studies are required to more explicitly characterize potential ecological differences among these cold-adapted candidate species, ‘A,’ ‘B,’ and ‘C.’ However, the phylogenetic PCA, corroborated these results, with temperature (BIOCLIM variables 1–3, 5–11), precipitation (BIOCLIM variables 12–19), and seasonality (BIOCLIM variables 4 and 7) generally correlating with candidate species-level lineages circumscribed here (**Supplementary Figure S2**).

**FIGURE 5:**
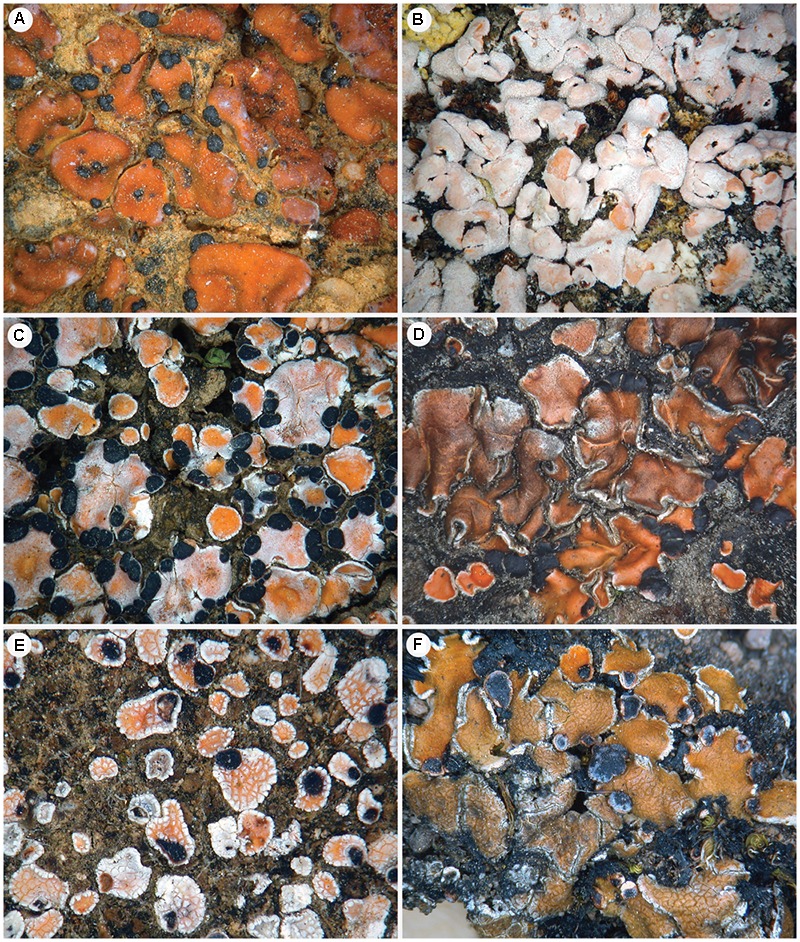
Morphological variation in the *P. crenata* s. lat. and *P. decipiens* s. lat. group. **(A)** *P. crenata* from the North Cape of South Africa (specimen ‘PS87’ [F268261 - S]). **(B)** *P. saviczii* from Spain, clade ‘Fb’ (specimen ‘PS46’ [F283532 - S]). **(C)** *P. decipiens* s. lat. from Spain, clade ‘D’ (specimen ‘PS09’ [Westberg SCIN077 - S]); **(D)** *P. decipiens* s. lat. from Austria, clade ‘C’ (specimen ‘PS12’ [Westberg HOCH042 - S]). **(E)** *P. decipiens* s. lat. from Spain, clade ‘Ga’ (specimen ‘PS06’ [Westberg SCIN071 -S]). **(F)** *P. decipiens* s. lat. from Sweden, clade ‘Gb’ (specimen ‘PS19’ [Westberg GYN051 - S]).

The remaining candidate species representing *P. decipiens* s. lat. – ‘D,’ ‘E,’ ‘Fa,’ ‘Fb’ (*P. saviczii*), ‘Fc,’ ‘Gb,’ and ‘Gc’ – are found in warmer, drier habitats (**Table 1** and **Supplementary Figure S1**). Clades ‘D,’ ‘Fc,’ and ‘Gb’ correspond to the ‘Spain,’ ‘Germany,’ and ‘Germany/Sweden’ clades, respectively, in Williams et al. (2017; **Figure 5**). In North America, the majority of the species-level lineages occur on calciferous soil in open habitats ranging from deserts to coniferous forests. Similarly, these lineages are found widely distributed in Asia, Europe, and Central Asia, with the exception of clade ‘E,’ which occurs exclusively in Australia. Additional sampling, coupled with ecological niche modeling (Austin, 2007), will likely provide important insights into the role of ecological specialization in the *P. decipiens* group. In addition to looking at bioclimatic variables that are commonly used to infer niche envelopes (Araújo and Peterson, 2012), investigating the role of physical properties of substrates may provide important insight into differences in distributions among these candidate species. *P. decipiens* s. lat. generally occurs generally on calciferous to gypsiferous soils, including *P. saviczii* (clade ‘Fb’) which occurs exclusively on highly gypsiferous soils and may co-occur with other *P. decipiens* s. lat. lineages. Whether differences in soil properties are related to species distributions in the *P. decipiens* groups remains untested. Availability of suitable photosynthetic algae (photobionts) may be another factor influencing the distribution of these lineages. Although previous studies suggested high photobiont diversity associating with the *P. decipiens* group (Ruprecht et al., 2014), a more recent study suggests that *P. decipiens* s. lat. may associate with a much narrower range of photobionts than previously thought (Williams et al., 2017). Additional research is required to elucidate the role of selection and specificity in structuring *P. decipiens* distributions.

### Taxonomic Implications

While both *P. crenata* and *P. decipiens* were traditionally thought to be widespread taxa within broad, intercontinental distributions (Timdal, 1984), our results show that a number of species-level lineages have been masked within these nominal taxa. Based on our results, specimens identified as *P. crenata* are clearly distinct from *P. decipiens* s. lat. However, what has traditionally been considered *P. crenata*, likely comprises at least three species-level lineages with distinct biogeography distributions – western North America, South Africa, and the Middle East (**Figure 2**). Given the deep divergence among these lineages and distinct phylogeographic patterns, there is convincing evidence that these represent valid species. While our current sampling did not include Australian populations of *P. crenata*, the chemistry in specimens in Australia and South Africa is highly variable. Future work investigating anatomical and morphological features, chemistry, and additional specimen sampling will be important to resolve the taxonomy and evolutionary relationships within this nominal taxon. In the meantime, we do not plan to make any formal taxonomic revisions for *P. crenata* s. lat., but encourage additional research into this group. Furthermore, we emphasize the importance of future studies to investigate patterns of gene flow and/or genetic population structure in this nominal taxon in order to better understand the evolutionary independence of these biogeographically structured lineages.

Similar to *P. crenata*, we also present convincing evidence that *P. decipiens* is comprised of up to 10 species-level lineages, some of which have distinct geographic and ecological distributions (Williams et al., 2017). However, pending detailed ecological and morphological studies of these candidate species, we refrain from making any formal taxonomic recommendations. In spite of the high degree of morphological variability in *P. crenata* s. lat. and *P. decipiens* s. lat. populations (**Figure 5**), exploratory morphological and chemical comparisons failed to reveal fixed diagnostic characters separating species-level lineages circumscribed here. Further complicating formal taxonomic revisions in *P. crenata* and *P. decipiens* is the fact that more than 10 epithets are available (Zahlbruckner, 1934), and linking candidate species circumscribed here with available epithets is beyond the scope of this project.

Our results reveal that a simplistic approach using the presence or absence of distinct secondary metabolites will fail to distinguish species for most groups circumscribed here, as a number of lineages were found to be chemically polymorphic and or included specimens in which no extrolites were detected (**Table 1**). In a few cases, such as the hyposalazinic/hypostictic acid chemotypes in *P. decipiens* s. lat. clade ‘Fc,’ distinct secondary metabolites appear to be restricted to specific species-level clades, although some acid-deficient specimens were also recovered in this clade.

Future investigations into the candidate species circumscribed in this study should be complemented with other lines of evidence in an integrative taxonomic approach (Dayrat, 2005; Fujita et al., 2012). From an evolutionary perspective, is not prerequisite that observable morphological differences coincide with evolutionarily independent lineages, although phenotypic traits have traditionally been used as proxies to infer evolutionary independence. In some cases, corroborating phenotypic characters have supported lichen-forming fungal species circumscribed using molecular sequence data (Divakar et al., 2005; Schneider et al., 2016), but in others, no diagnostic phenotypic traits segregating species have been observed.

### Potential Directions for Future Studies

Global aridification and development of open habitats during the Neogene have been proposed to play important roles in diversification of lichen-forming fungal components of BSC. However, in spite of the fact that Australia includes one of the largest arid biomes in the world, much less is known regarding the timing and potential factors influencing diversification of symbiotic fungi in Australia in general (Amo de Paz et al., 2012). Additional studies investigating the temporal component of fungal diversification in Australia, especially in comparison to other arid regions, will likely provide key insights into the processes of diversification in this unique continent. Specifically, studying other groups of lichenized fungi will provide insight into whether or not there are similar underlying factors that have shaped fungal diversity in Australia.

Southern Africa is another fascinating region in terms of lichen biogeography (Hale, 1990). In this study, striking phylogenetic diversity was revealed in the nominal taxon *P. crenata*, with at least three candidate species revealed in South Africa alone. However, we were unable to sample populations representing *P. decipiens* from South Africa, and the relationship of these populations with *P. crenata* populations, and other *P. decipiens*, remains unknown. *P. crenata* and *P. decipiens* are morphological variable in South Africa, and intermediates forms not easily identifiable to either taxa are commonly encountered. Additional collections from South Africa will be required to elucidate the relationships of these populations to the lineages recovered in this study. Based on our limited sampling of *Psora* populations, it appears that intercontinental dispersal and long-distance dispersal may be less frequent in the Southern hemisphere when compared to similar populations in the Northern Hemisphere (e.g., North America, Europe, and Central Asia). Additional research will be required to adequately test this hypothesis and assess potential limitations to dispersal.

The relationship of *P. decipiens* populations in a number of other major regions have not yet been assessed within a molecular phylogenetic context, including South America (Peru), Tibet Plateau/Himalayas, Middle East, and Central Asia (**Figure 1**). Although *P. saviczii* (clade ‘Fb’) is known from a relatively small geographic distribution in hot, dry, gypsiferous habitats in Spain and Russia, and this taxon was represented by only four specimens. Sampling additional populations throughout the species range will be essential to confirm that *P. saviczii* is, in fact, monophyletic.

Our sampling of *P. crenata* was relatively sparse in comparison to *P. decipiens*. Representing additional populations throughout the range of this taxon will likely reveal novel biogeographic insight and may influence subsequent phylogenetic inference and interpretations.

## Author Contributions

SL and MarW designed and implemented this study. MN, LS, LW, MatW, ET, and HL made substantial contributions to the conceptual design of this work. SL, MarW, MS, JE, ET, LS, and MatW were central to the acquisition of specimens and other data. SL led the analytical component of this study, and all co-authors made substantial contributions to the interpretation of data. SL drafted the manuscript, with significant contributions from all co-authors.

## Conflict of Interest Statement

The authors declare that the research was conducted in the absence of any commercial or financial relationships that could be construed as a potential conflict of interest.

## Footnotes

1 http://www.phylo.org/portal2/

2 http://www.phylo.org/portal2/

## Supplementary Material

FIGURE S1 Bioclimatic data for 19 Bioclim variables for candidate species. Latitude and longitude were used to extract bioclimatic variables at a spatial resolution of 2.5 min, using the R (R Core Team, 2015) package raster (Hijmans et al., 2015).

FIGURE S2 Niche ecospace as described by PC1 and PC2, derived from a phylogenetic PCA on the mean candidate species values for 19 bioclim variables. Candidate species are plotted by name, and eigenvectors as arrows. Together, PC1 (66.4%) and PC2 (20.2%) account for account for >86% of the variation. Temperature and precipitation variables are associated with PC1, while temperature seasonality and annual range are associated with PC2. Abbreviations for individual bioclim variables correspond to the following: *bio1* = Annual Mean Temperature; *bio2* = Mean Diurnal Range [Mean of monthly (max temperature - min temperature)]; *bio3* = Isothermality (bio2/bio7) (^∗^ 100); *bio4* = Temperature Seasonality (standard deviation ^∗^100); *bio5* = Max Temperature of Warmest Month; *bio6* = Min Temperature of Coldest Month; *bio7* = Temperature Annual Range (bio5-bio6); *bio8* = Mean Temperature of Wettest Quarter; *bio9* = Mean Temperature of Driest Quarter; *bio10* = Mean Temperature of Warmest Quarter; *bio11* = Mean Temperature of Coldest Quarter; *bio12* = Annual Precipitation; *bio13* = Precipitation of Wettest Month; *bio14* = Precipitation of Driest Month; *bio15* = Precipitation Seasonality (Coefficient of Variation); *bio16* = Precipitation of Wettest Quarter; *bio17* = Precipitation of Driest Quarter; *bio18* = Precipitation of Warmest Quarter; *bio19* = Precipitation of Coldest Quarter.

TABLE S1 List of specimens included in this study.
