# Supplementary material for: Multiple, Distinct Intercontinental Lineages but Isolation of Australian Populations in a Cosmopolitan Lichen-Forming Fungal Taxon, Psora decipiens (Psoraceae, Ascomycota)
Source: Front Microbiol. 2018 Feb 23;9:283. doi: 10.3389/fmicb.2018.00283 (PMC5829036; doi:10.3389/fmicb.2018.00283)

Supplementary Fig. S1.

BIO1 - Annual Mean Temperature

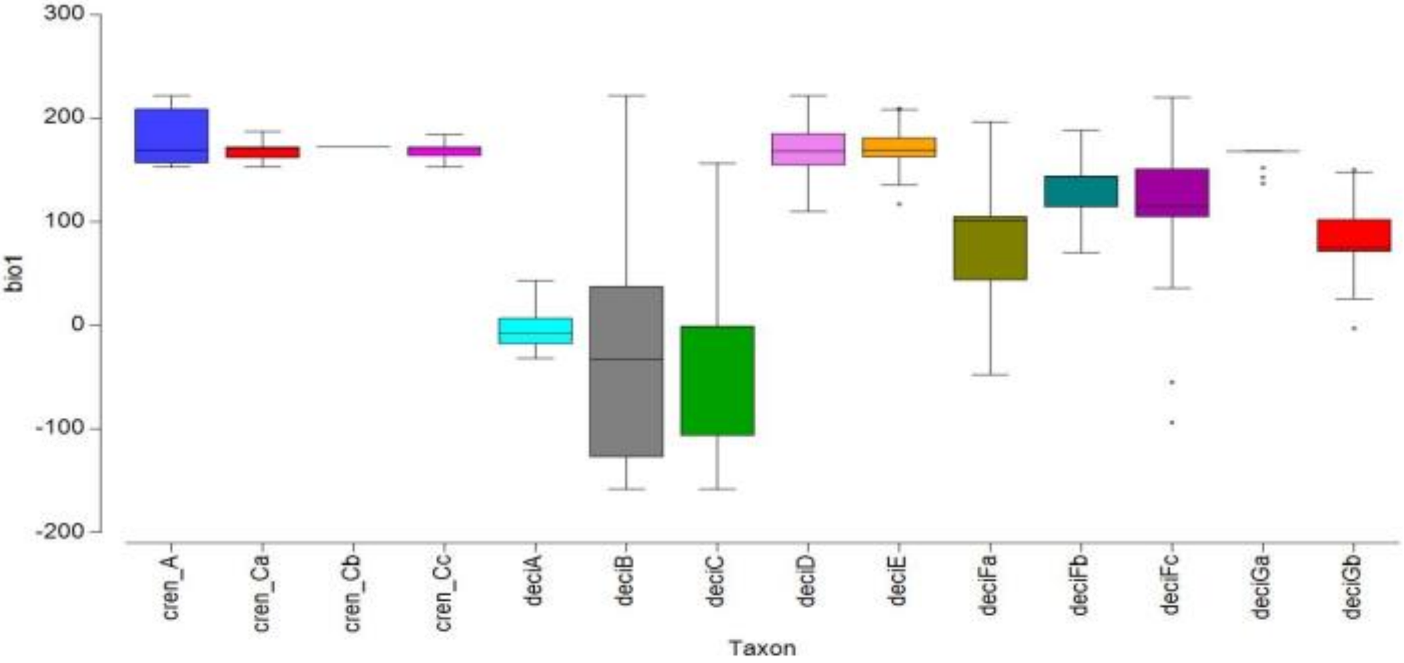

BIO2 - Mean Diurnal Range (Mean of monthly (max temp - min temp))

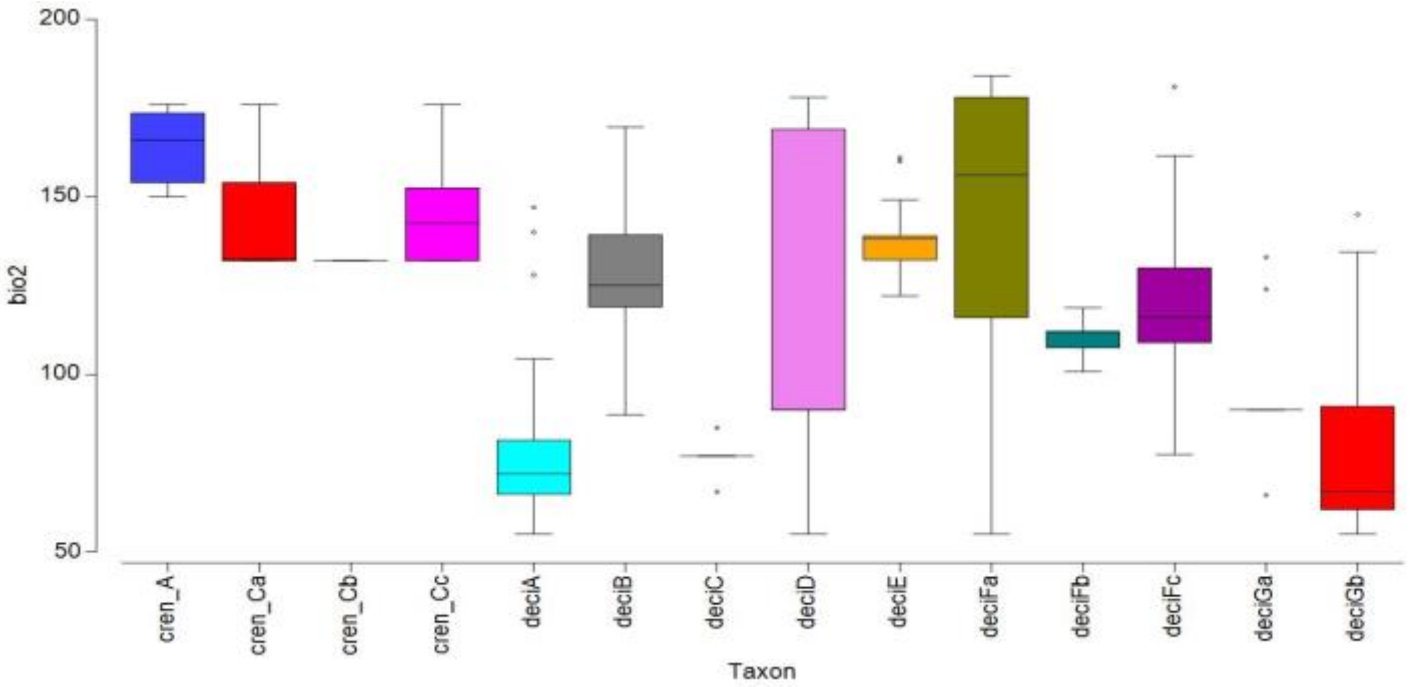

BIO3 - Isothermality (BIO2/BIO7) (* 100)

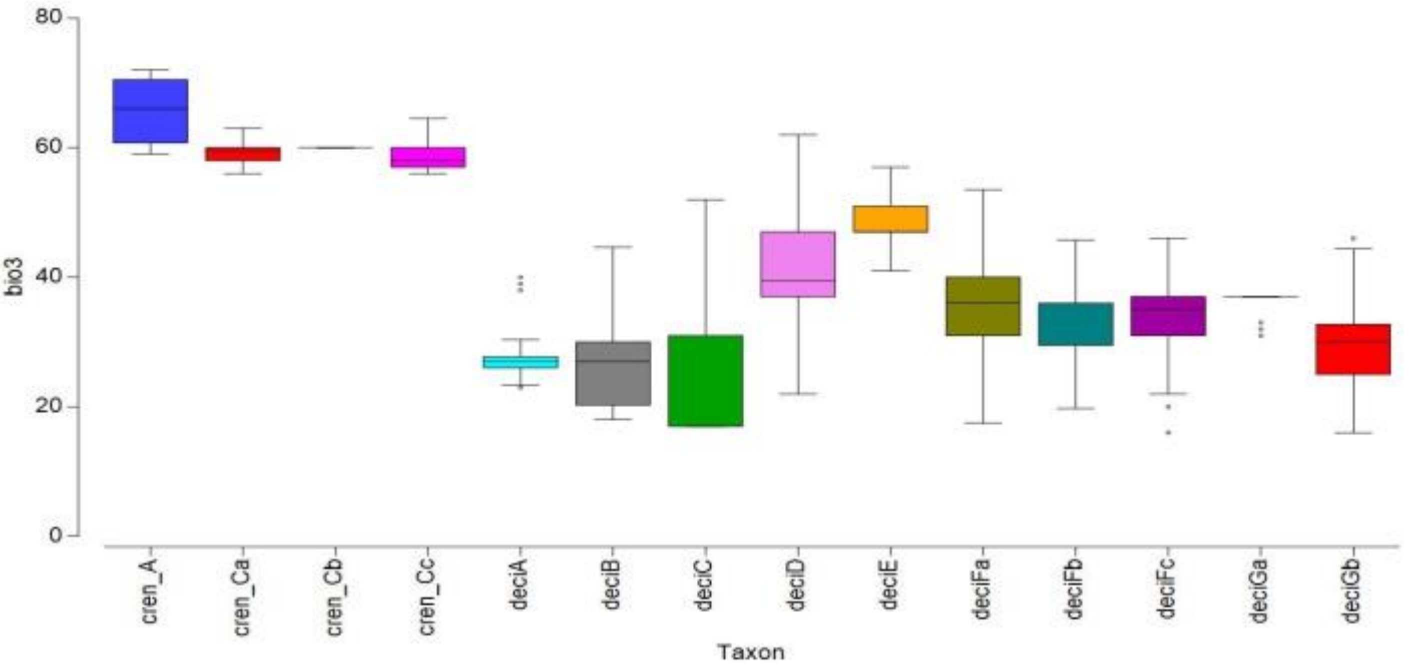

BIO4 - Temperature Seasonality (standard deviation *100)

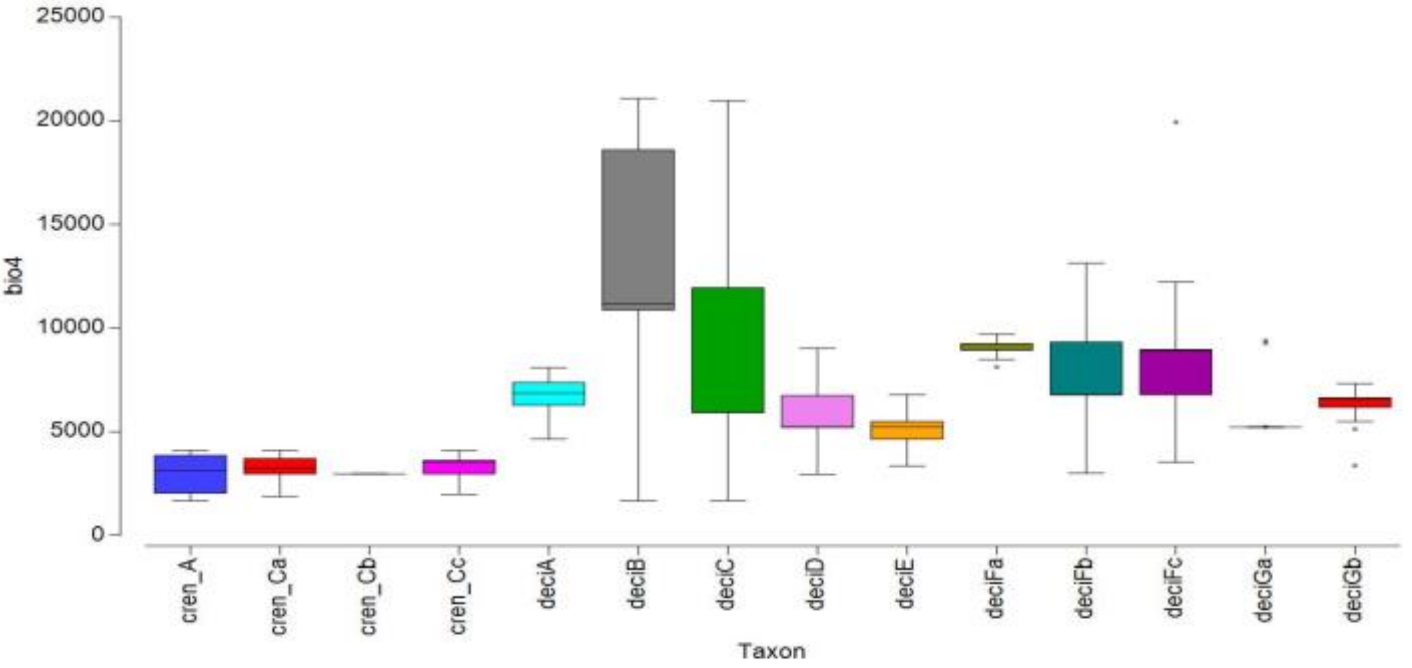

BIO5 - Max Temperature of Warmest Month

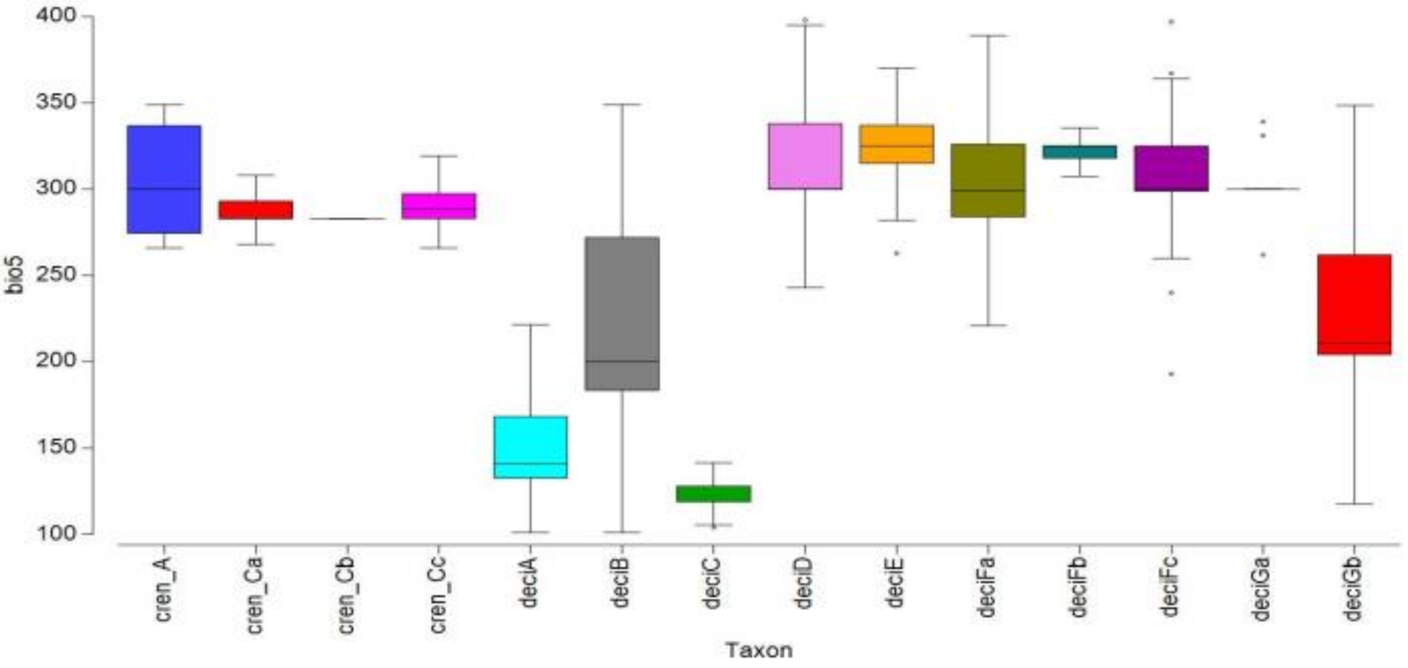

BIO6 - Min Temperature of Coldest Month

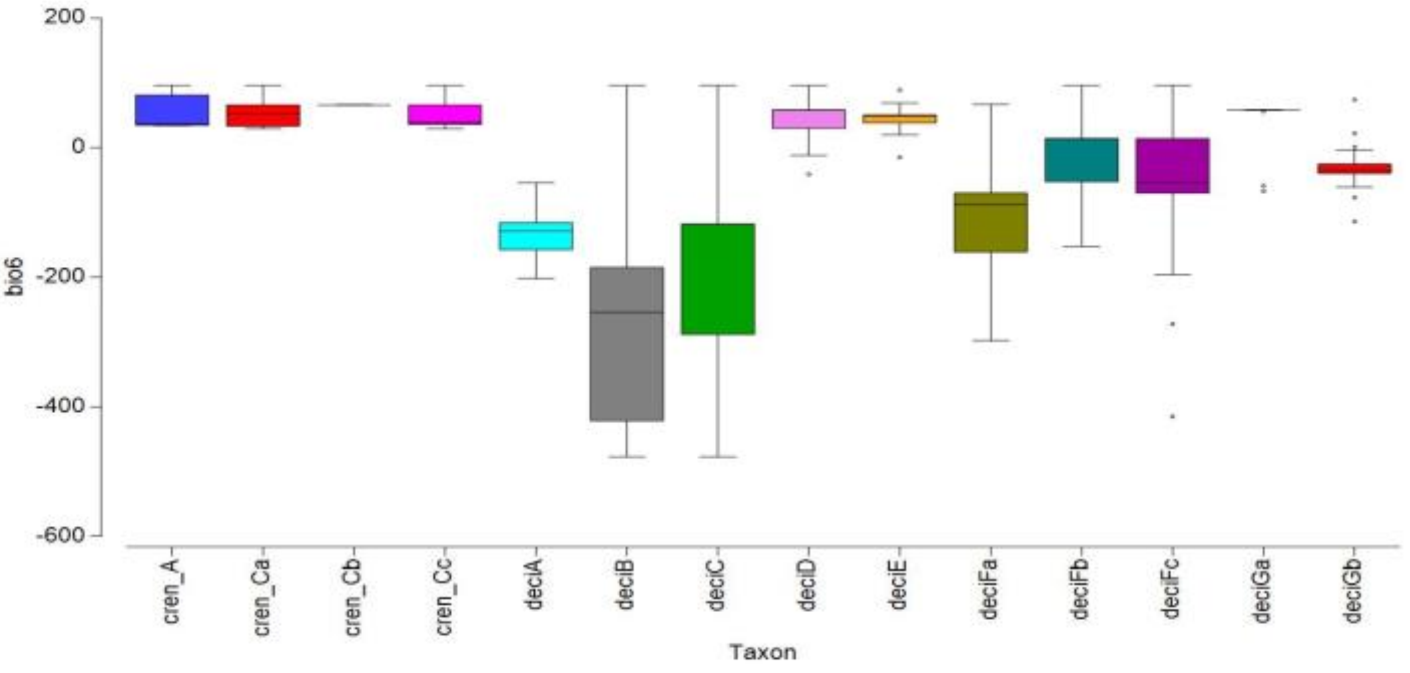

BIO7 - Temperature Annual Range (BIO5-BIO6)

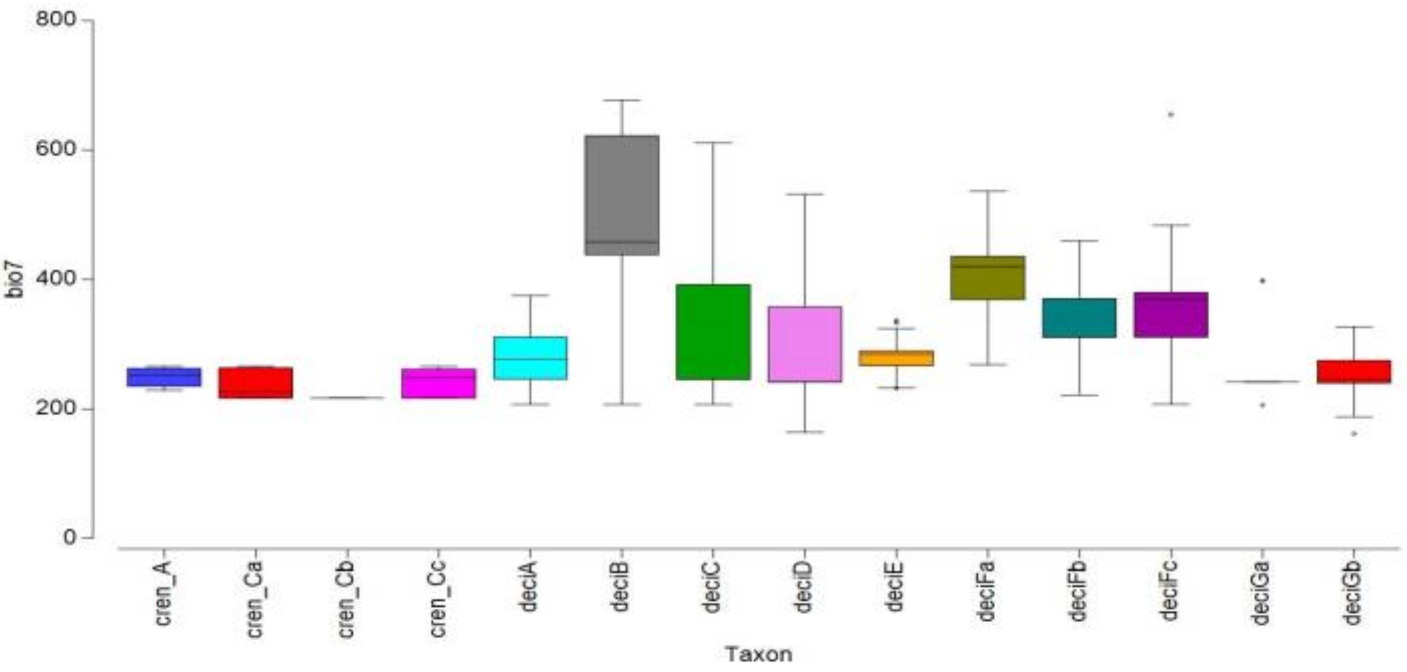

BIO8 - Mean Temperature of Wettest Quarter

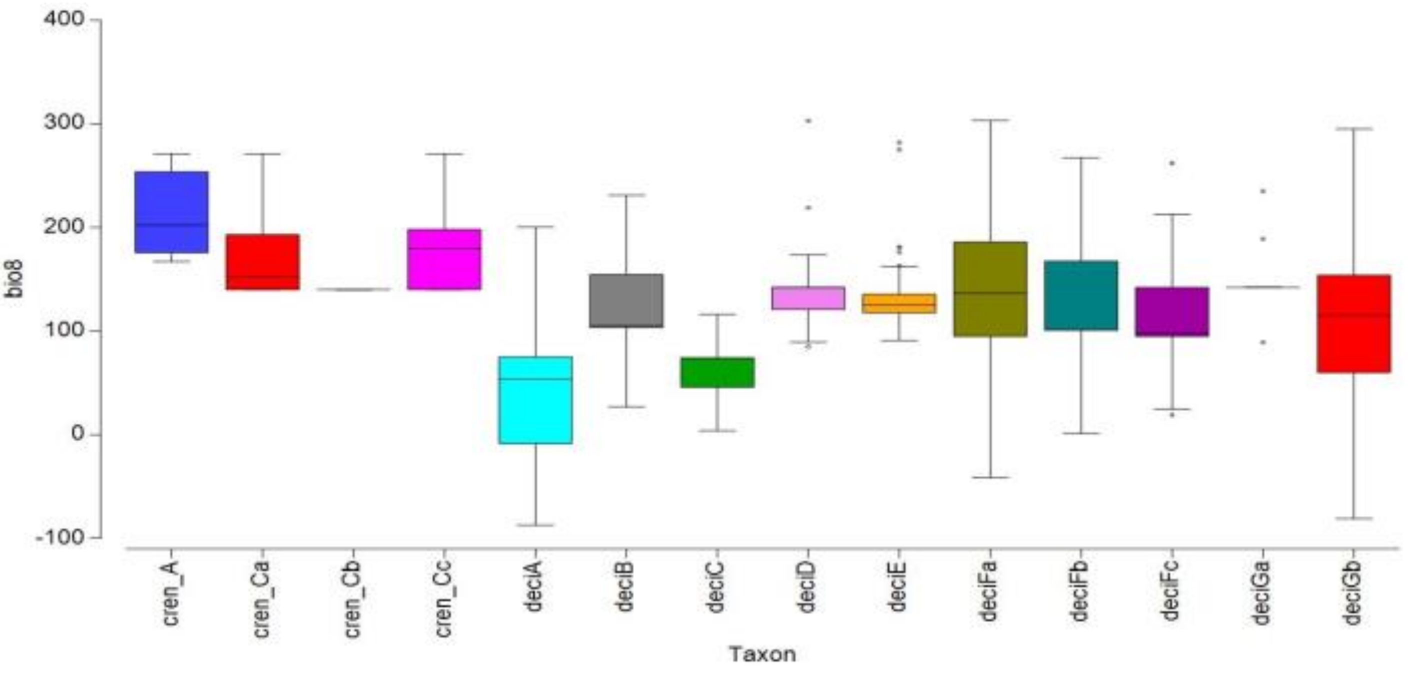

BIO9 - Mean Temperature of Driest Quarter

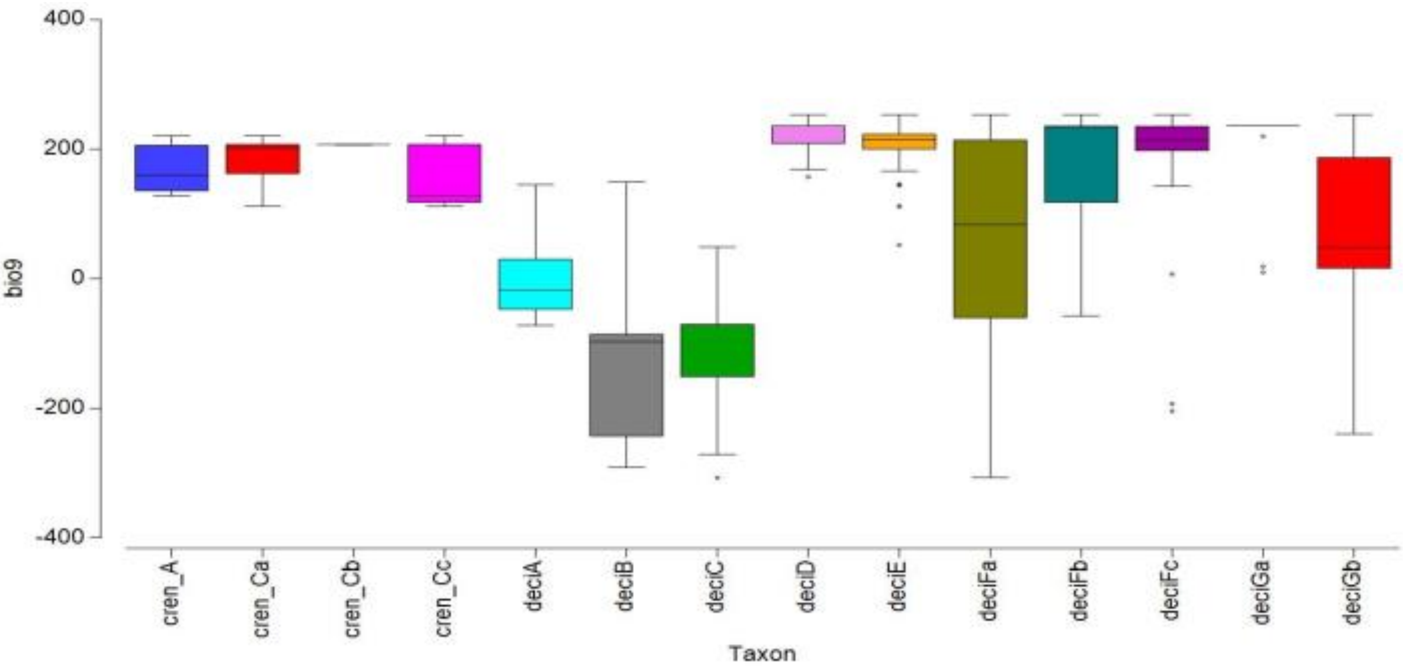

BIO10 - Mean Temperature of Warmest Quarter

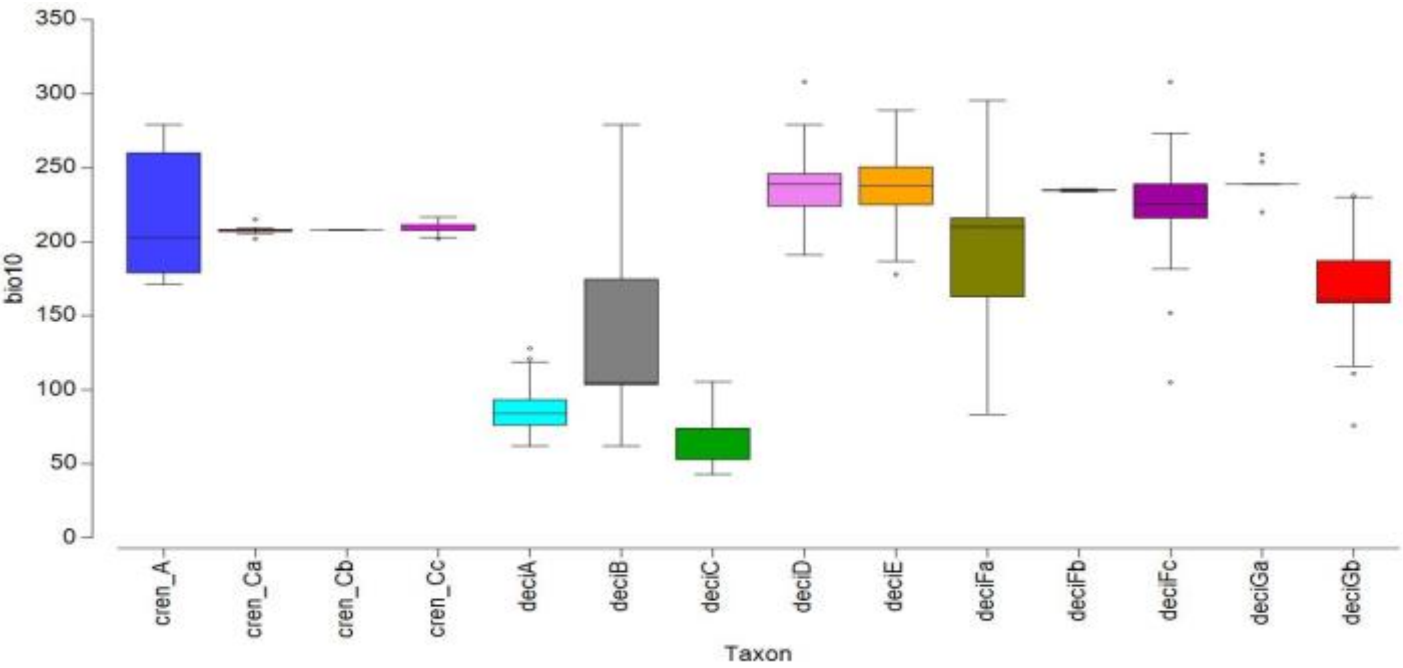

BIO11 1 Mean Temperature of Coldest Quarter

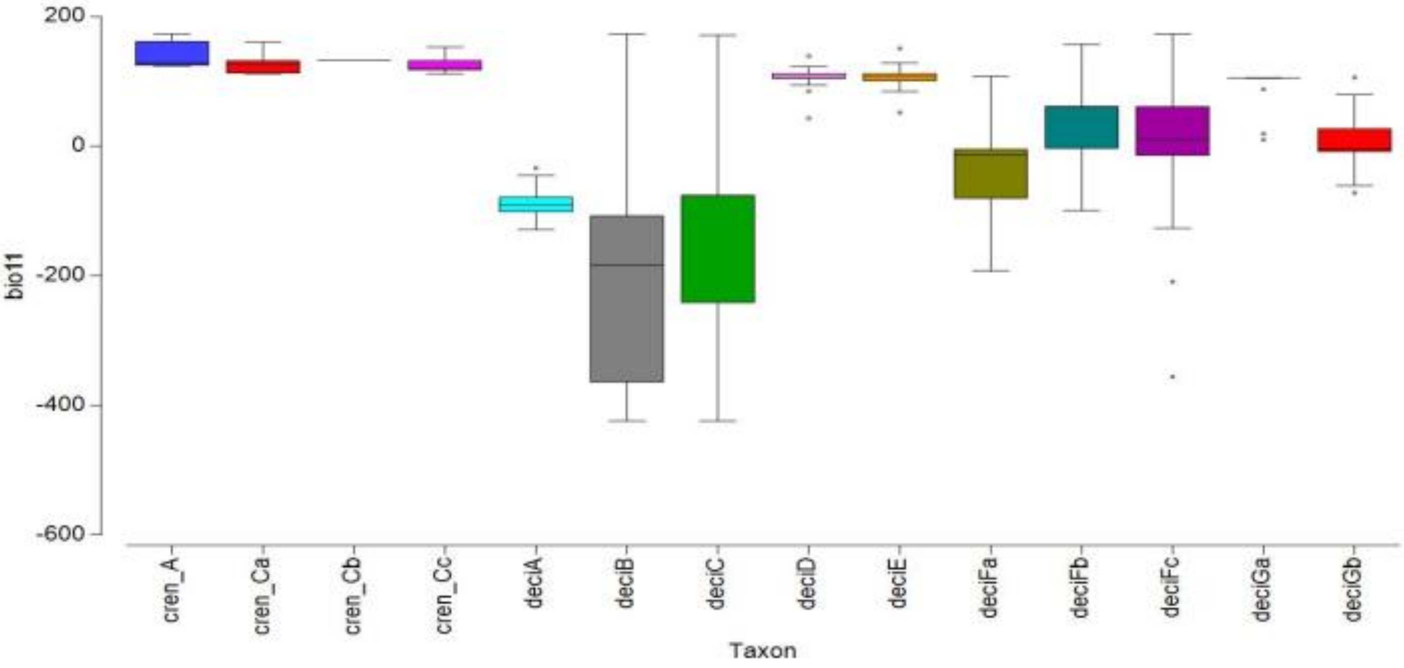

BIO12 - Annual Precipitation

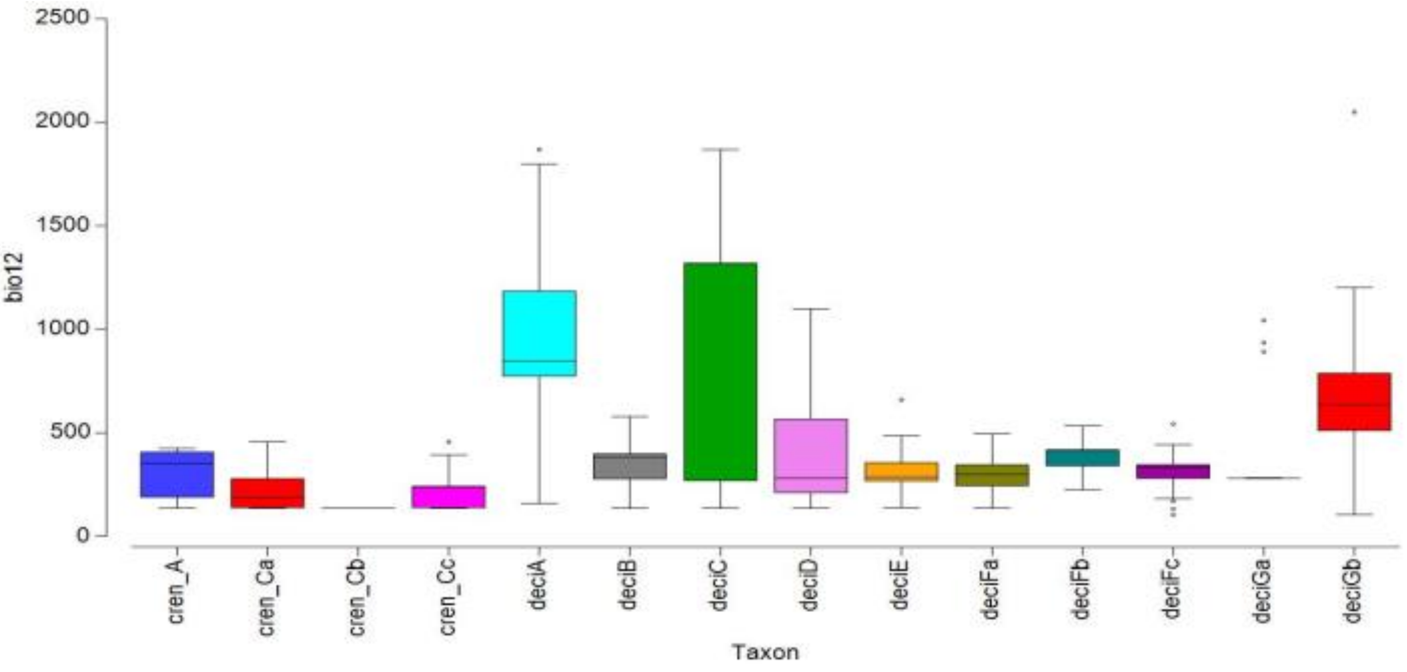

BIO13 - Precipitation of Wettest Month

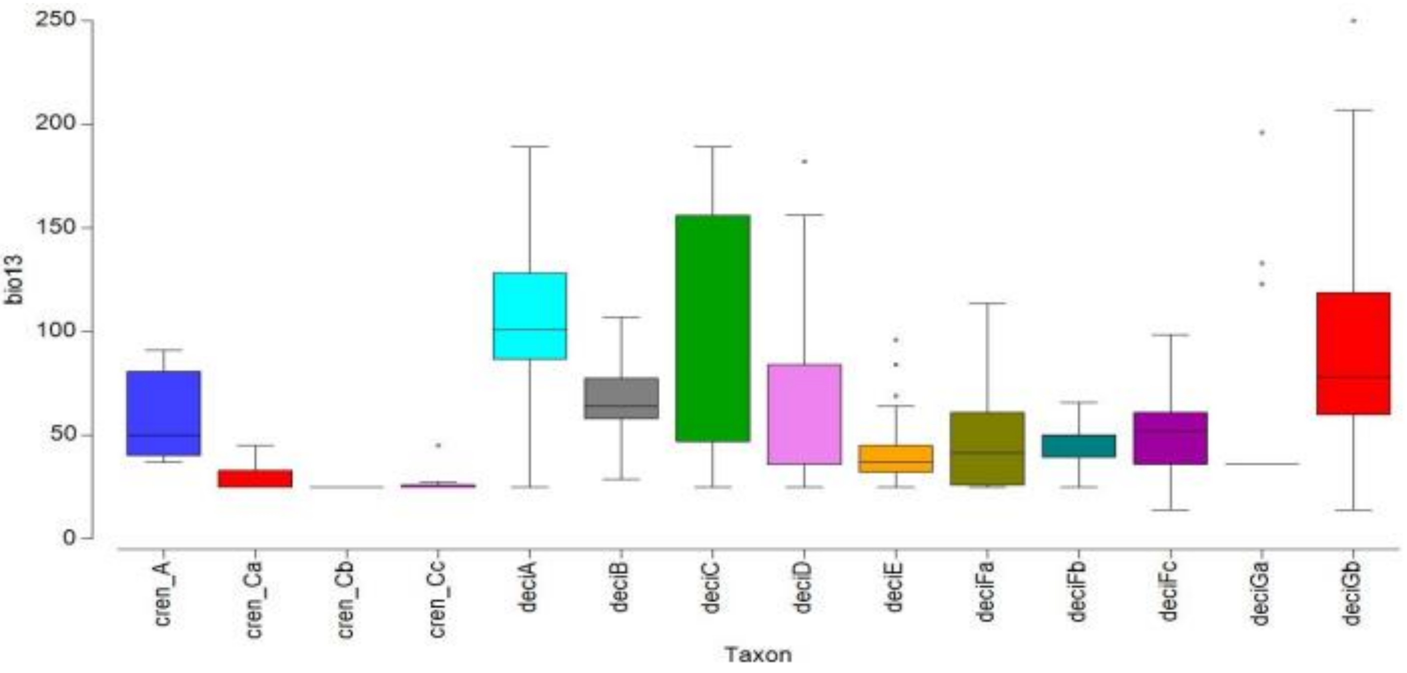

BIO14 – Precipitation of Driest Month

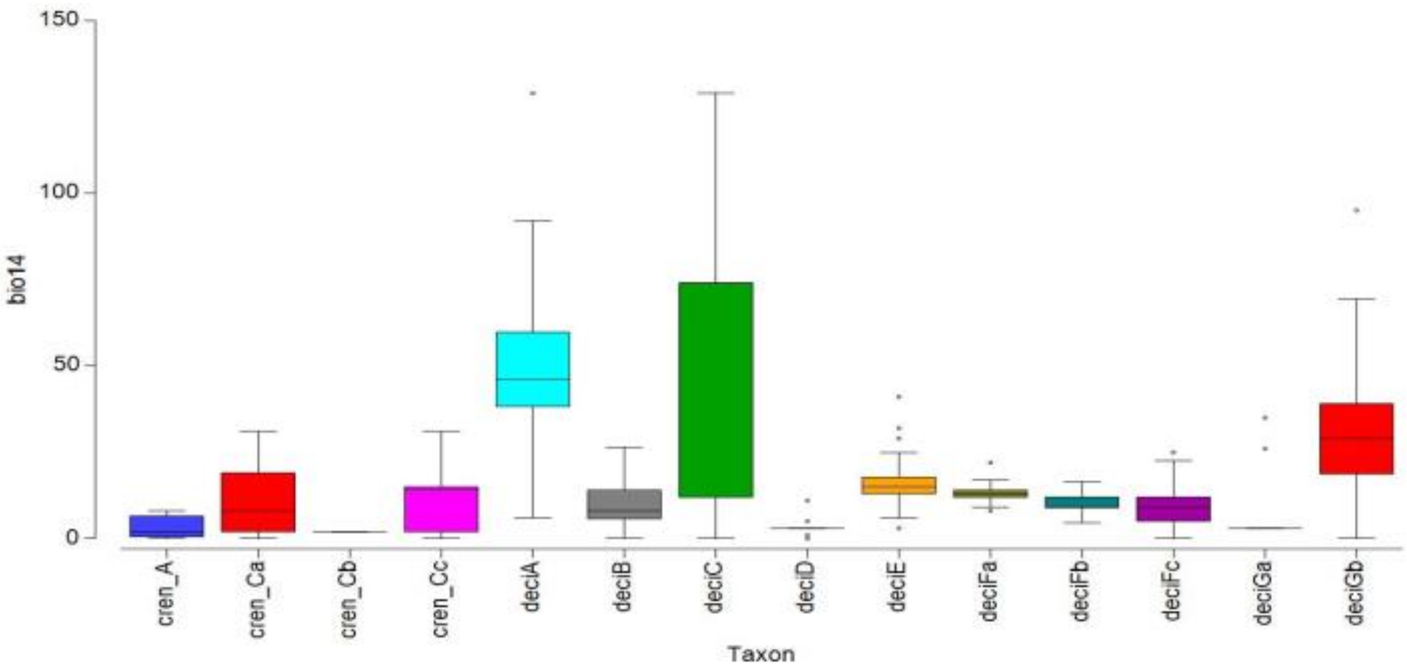

BIO15 - Precipitation Seasonality (Coefficient of Variation)

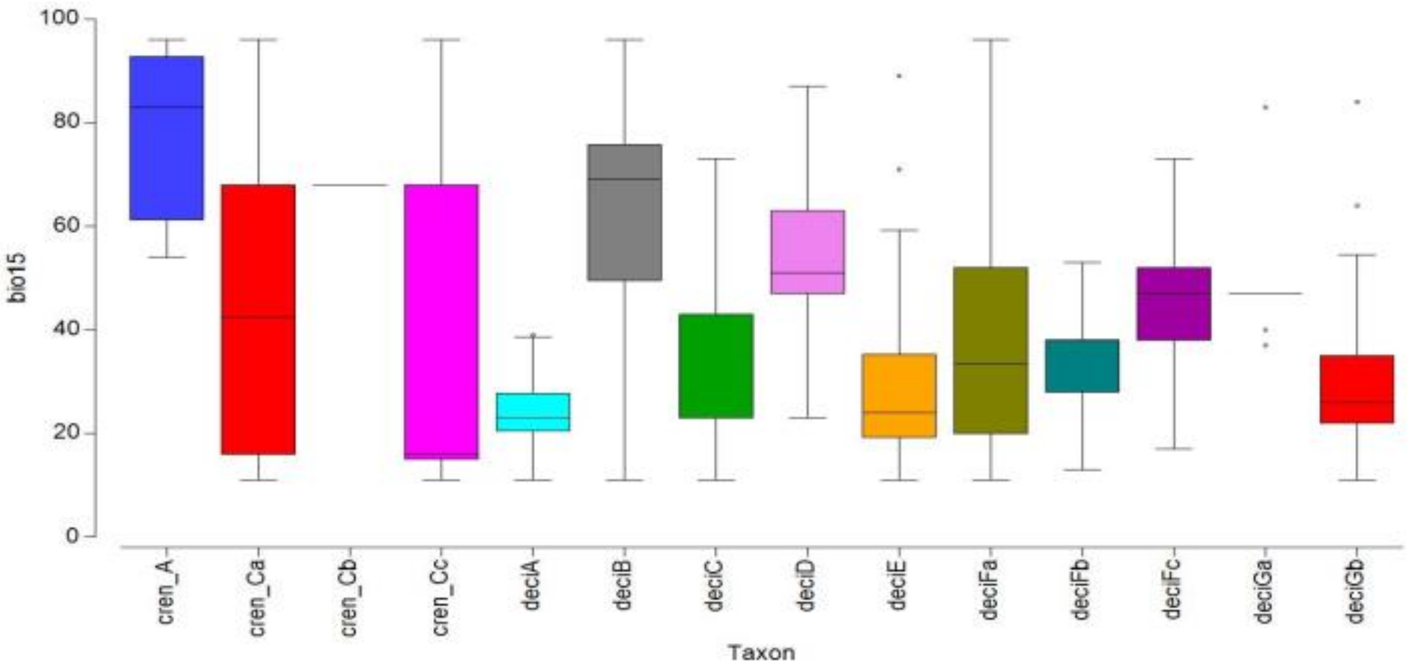

BIO16 = Precipitation of Wettest Quarter

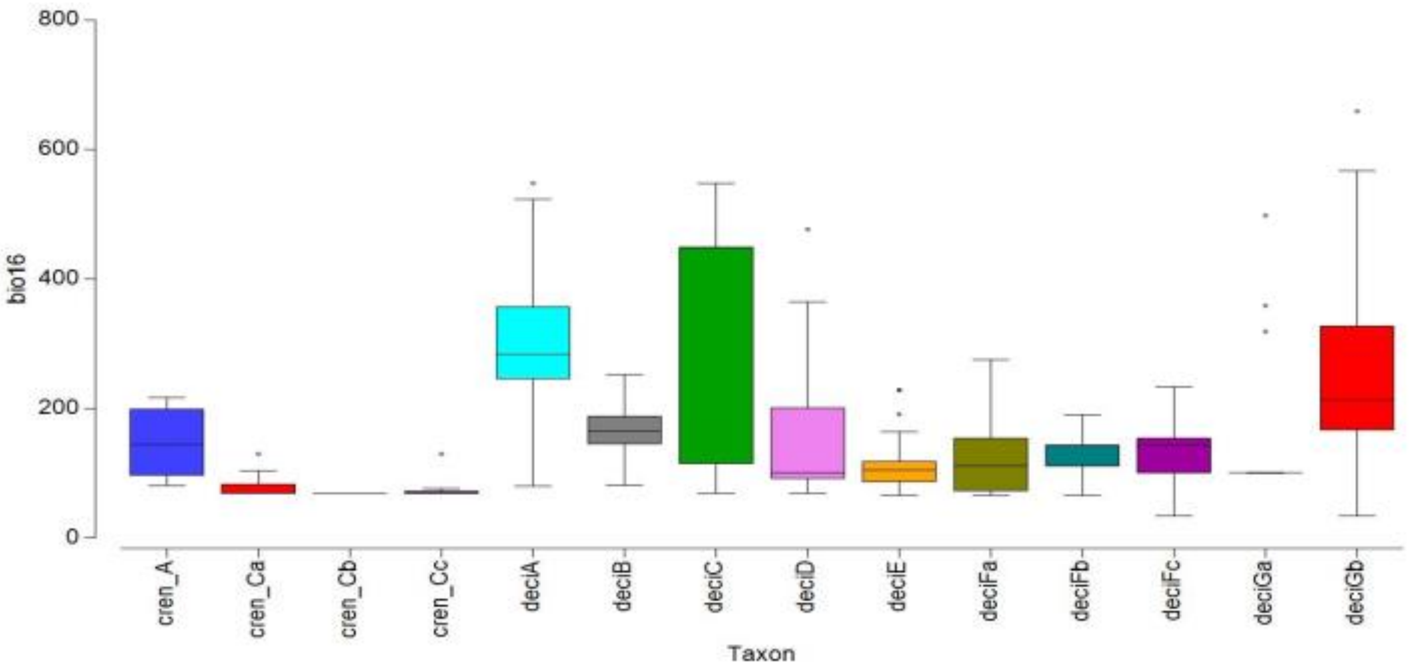

BIO17 - Precipitation of Driest Quarter

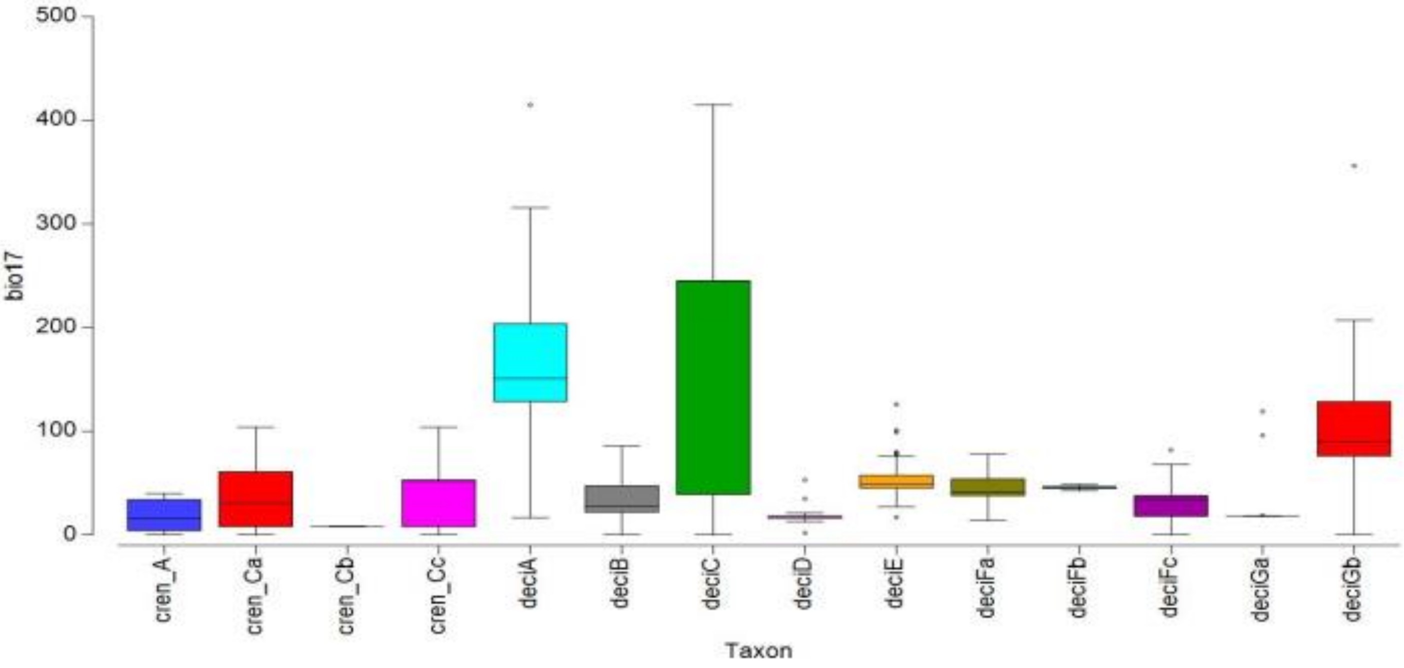

BIO18 = Precipitation of Warmest Quarter

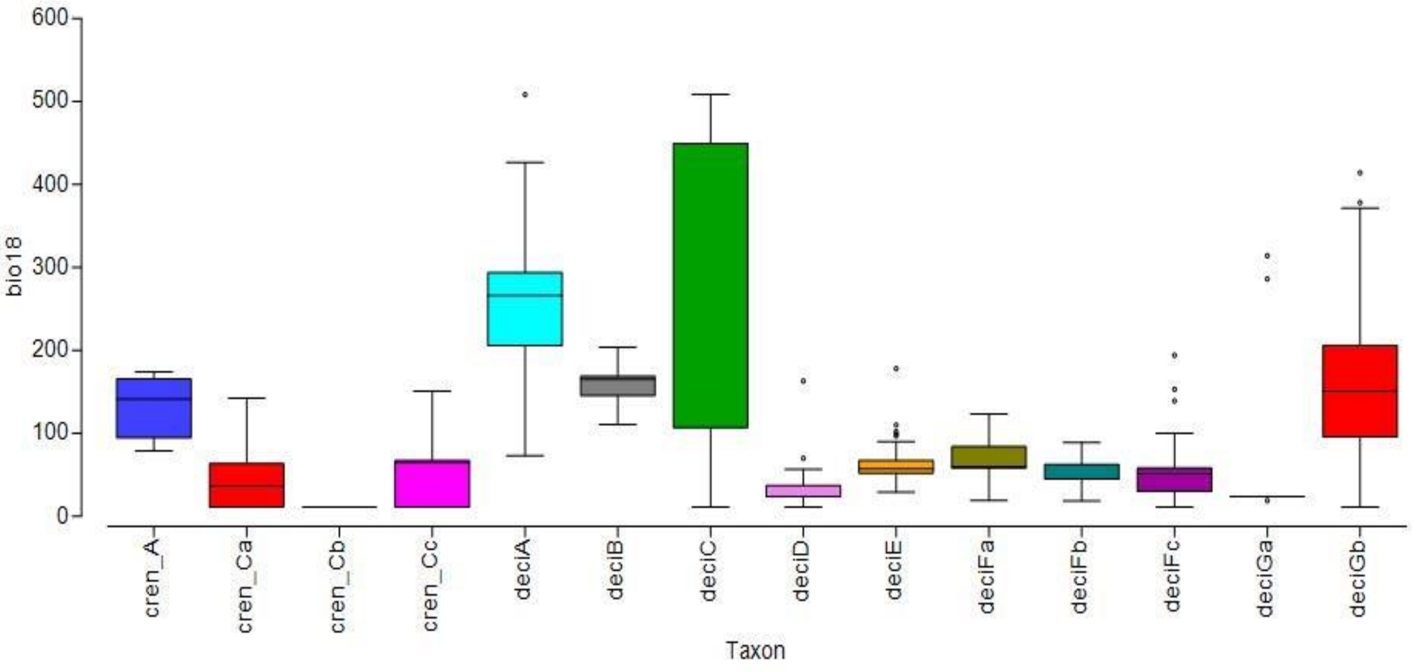

BIO19 - Precipitation of Coldest Quarter

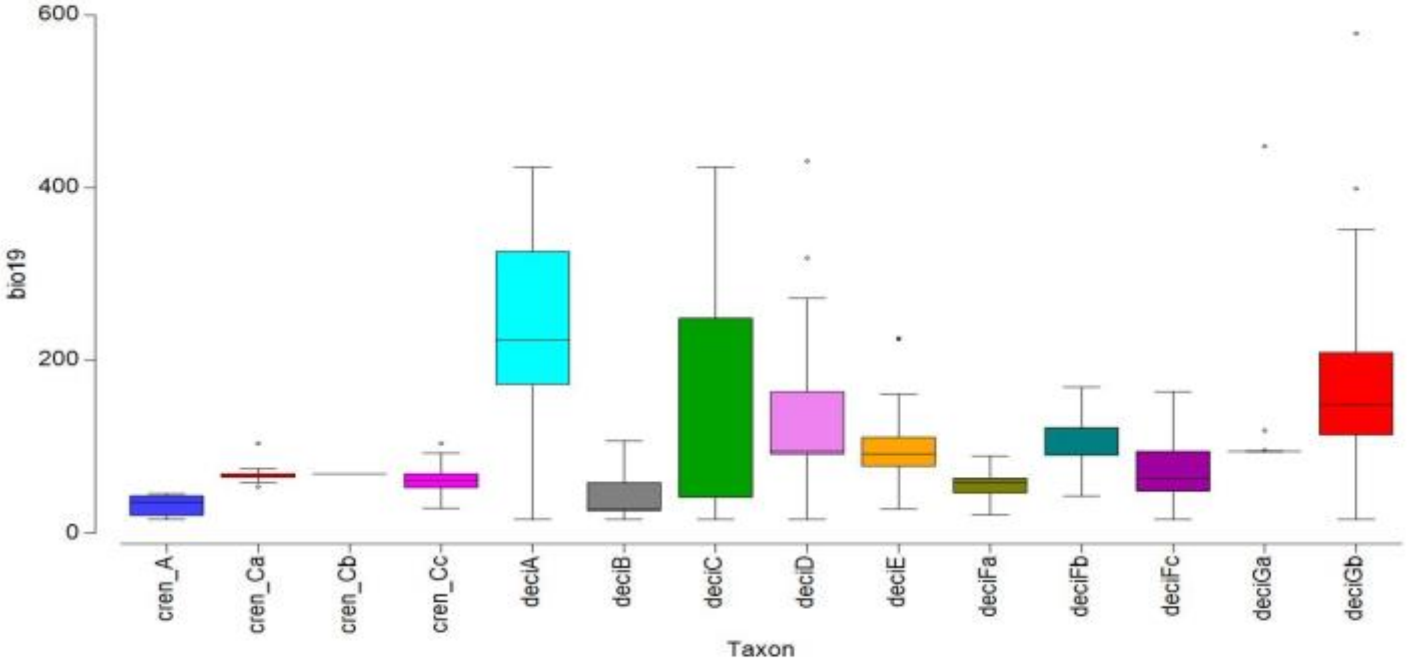

Supplement: FIGURE S1 — Bioclimatic data for 19 Bioclim variables for candidate species. Latitude and longitude were used to extract bioclimatic variables at a spatial resolution of 2.5 min, using the R (R Core Team, 2015) package raster (Hijmans et al., 2015). [file Image_1.PDF]
