# Supplementary figures and images for: Multiple, Distinct Intercontinental Lineages but Isolation of Australian Populations in a Cosmopolitan Lichen-Forming Fungal Taxon, Psora decipiens (Psoraceae, Ascomycota)
Source: Front Microbiol. 2018 Feb 23;9:283. doi: 10.3389/fmicb.2018.00283 (PMC5829036; doi:10.3389/fmicb.2018.00283)

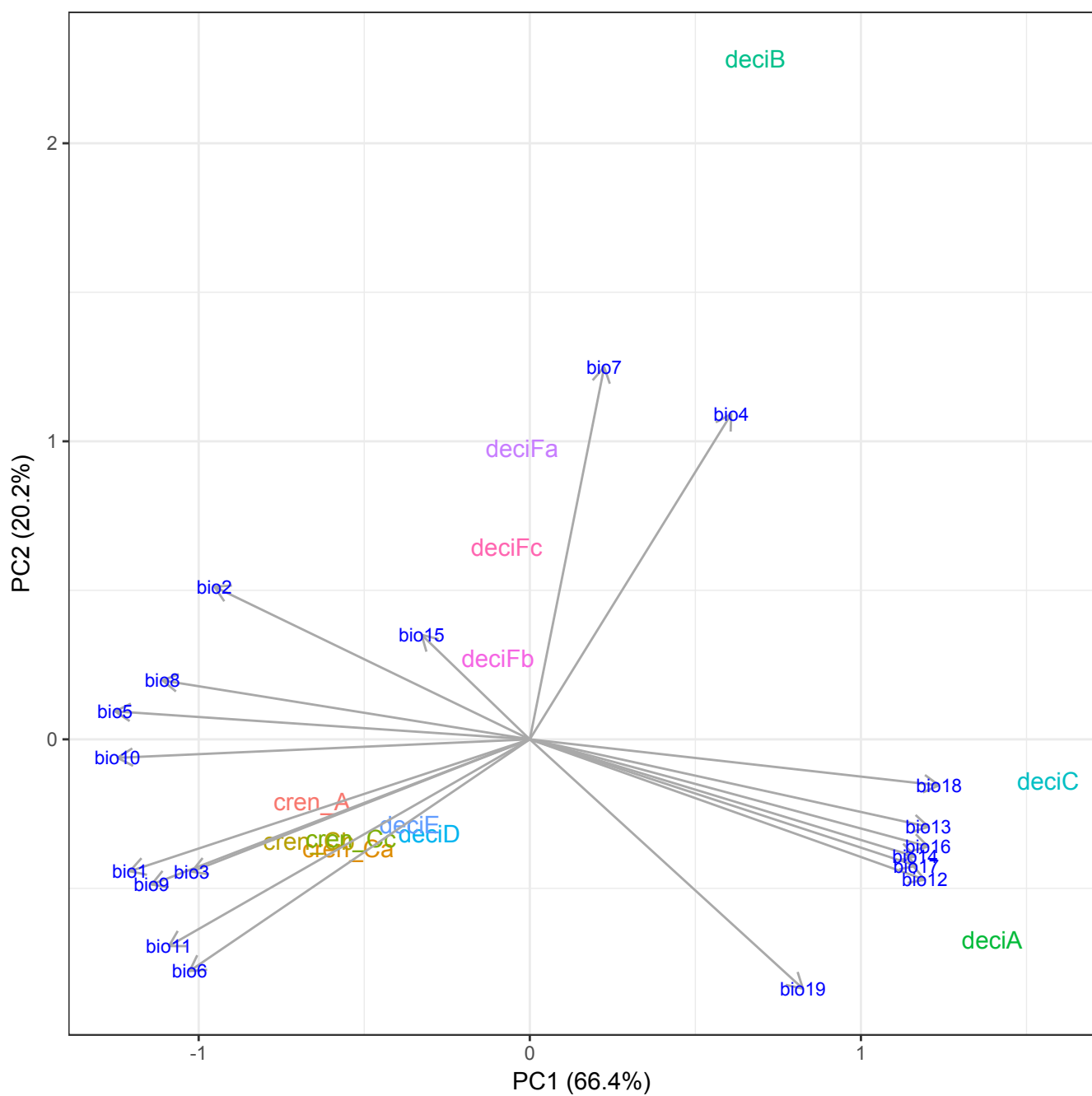

Supplement: FIGURE S2 — Niche ecospace as described by PC1 and PC2, derived from a phylogenetic PCA on the mean candidate species values for 19 bioclim variables. Candidate species are plotted by name, and eigenvectors as arrows. Together, PC1 (66.4%) and PC2 (20.2%) account for account for >86% of the variation. Temperature and precipitation variables are associated with PC1, while temperature seasonality and annual range are associated with PC2. Abbreviations for individual bioclim variables correspond to the following: bio1 = Annual Mean Temperature; bio2 = Mean Diurnal Range [Mean of monthly (max temperature - min temperature)]; bio3 = Isothermality (bio2/bio7) (∗ 100); bio4 = Temperature Seasonality (standard deviation ∗100); bio5 = Max Temperature of Warmest Month; bio6 = Min Temperature of Coldest Month; bio7 = Temperature Annual Range (bio5-bio6); bio8 = Mean Temperature of Wettest Quarter; bio9 = Mean Temperature of Driest Quarter; bio10 = Mean Temperature of Warmest Quarter; bio11 = Mean Temperature of Coldest Quarter; bio12 = Annual Precipitation; bio13 = Precipitation of Wettest Month; bio14 = Precipitation of Driest Month; bio15 = Precipitation Seasonality (Coefficient of Variation); bio16 = Precipitation of Wettest Quarter; bio17 = Precipitation of Driest Quarter; bio18 = Precipitation of Warmest Quarter; bio19 = Precipitation of Coldest Quarter. [file Image_2.PDF]
